# Multi-omics analysis reveals the mechanism of Huaganjian in alleviating cholestatic liver fibrosis

**DOI:** 10.3389/fphar.2026.1744312

**Published:** 2026-03-12

**Authors:** Zijun Zhang, Ahua Ku, Rong Ji, Binbin Song

**Affiliations:** Key Laboratory of Ethnomedicine (Minzu University of China), Ministry of Education, School of Pharmacy, Minzu University of China, Beijing, China

**Keywords:** cholestatic liver fibrosis, Huaganjian, metabolomics, microbiomics, transcriptomics

## Abstract

Huaganjian (HGJ) is a traditional Chinese medicinal formula with liver-protective effects. However, the pharmacological mechanisms of the effects of HGJ on cholestatic liver fibrosis (CLF) are yet to be clarified. To evaluate the effects of HGJ on CLF and elucidate the underlying mechanisms, C57BL/6J mice were fed a 0.1% 3, 5-diethoxycarbonyl-1, 4-dihydrocollidine (DDC) diet to induce CLF. The efficacy of HGJ was evaluated by measuring the biochemical indicators of liver function, fibrosis, and histology. The underlying mechanisms were investigated using an integrated multi-omics approach, including fecal 16S rRNA sequencing, serum metabolomics, and hepatic transcriptomic analysis. The findings were further validated using reverse transcription-quantitative polymerase chain reaction (RT-qPCR) and Western blotting (WB). HGJ significantly alleviated liver injury, cholestasis, and fibrosis. Microbiome analysis revealed that *Bifidobacterium, Turicibacter, and Clostridium_sensu_stricto_1* abundances were positively correlated with liver injury and fibrosis marker levels, and these abundances decreased following HGJ treatment. Metabolomic analysis identified 531 differential metabolites, including 299 upregulated and 232 downregulated metabolites, following HGJ intervention. Hepatic transcriptomic analysis revealed 164 differentially expressed genes, including 102 upregulated and 62 downregulated genes. Integrated multi-omics analysis revealed that HGJ alleviated CLF by modulating the glycine/serine/threonine metabolism pathway. RT-qPCR and Western blotting experiments confirmed that in this pathway, aminolevulinic acid synthase 1 levels decreased, whereas serine dehydratase and serine dehydratase-like levels increased after HGJ treatment. Overall, HGJ effectively alleviated CLF, and its mechanisms of action were closely linked to the regulation of the glycine/serine/threonine metabolism pathway.

## 1 Introduction

Cholestasis is a significant etiology of liver fibrosis. Persistent biliary stasis triggers chronic inflammatory responses, leading to hepatocyte damage and excessive extracellular matrix deposition, resulting in cholestatic liver fibrosis (CLF) (Wang et al., 2024). Early intervention can reverse liver fibrosis and prevent or delay its progression to cirrhosis or hepatocellular carcinoma (Roehlen et al., 2020). CLF tends to progress more rapidly to cirrhosis and hepatocellular carcinoma than non-cholestatic liver fibrosis. Current treatments for CLF, such as ursodeoxycholic acid and obeticholic acid (OCA), are limited by their efficacy and adverse event rates (Corpechot et al., 2020; Kowdley et al., 2020). Therefore, there is a crucial need to develop more effective treatments for CLF to inhibit its progression.

Huaganjian (HGJ), a traditional Chinese medicine formula attributed to the Ming Dynasty physician Zhang Jingyue, is documented in “Jingyue Quanshu.” It comprises the following herbs: *Citrus × aurantium* L., *Citrus reticulata* Blanco, *Paeonia lactiflora* Pall., *Paeonia × suffruticosa* Andrews, *Gardenia jasminoides* J. Ellis, *Alisma orientale* (Sam.) Juz, and *Fritillaria thunbergii* Miq. HGJ has traditionally been used to treat Liver Qi Stagnation. Preliminary evidence from animal models indicates its hepatoprotective effects against steatohepatitis and α-naphthylisothiocyanate-induced cholestasis (Dong et al., 2021; Dong et al., 2025). The therapeutic potential and underlying mechanisms of HGJ in CLF, a more advanced and clinically challenging pathological condition, remain unknown. This study aimed to systematically assess the anti-cholestatic and anti-fibrotic effects of HGJ in a CLF model and uncover its mechanisms of action through integrated multi-omics analyses.

Recent advances in multi-omics research have created new opportunities for identifying the active metabolites of herbal medicines and understanding their therapeutic mechanisms (Geng et al., 2021; Li Y. et al., 2025; Wan et al., 2025). Unlike traditional single-omics methods, integrated multi-omics strategies provide a comprehensive overview by revealing dynamic interactions within the “microbe-metabolite-gene” network, thus enabling the systematic exploration of disease mechanisms and therapeutic targets (Lv et al., 2024). Here, we established a classic CLF mouse model and evaluated the effects of HGJ (Ravichandra and Schwabe, 2021). We used an innovative tri-omics approach, combining 16S rRNA gene sequencing (gut microbiota), untargeted serum metabolomics, and hepatic transcriptomics, to investigate the mechanisms of action of HGJ against CLF.

## 2 Materials and methods

### 2.1 Preparation and analysis of HGJ extract

A mixture of 38.75 g *Citrus × aurantium* L. (Batch No. 210919003, Qiancao, Beijing, China), 38.75 g *C. reticulata* Blanco (Batch No. 23062002, Lvye, Beijing, China), 38.75 g *P. lactiflora* Pall. (Batch No. 23061201, Lvye, Beijing, China), 29.10 g *Paeonia × suffruticosa* Andrews (Batch No. 2303086, Xiangwei, Beijing, China), 29.10 g *G. jasminoides* J. Ellis (Batch No. 221019011, Qiancao, Beijing, China), 29.10 g *A. orientale* (Sam.) Juz. (Batch No. 22122501, Lvye, Beijing, China), and 43.62 g *F. thunbergii* Miq. (Batch No. 2207046, Zhoushi Shizhentang, Beijing, China) in 750 mL of distilled water was boiled for 2 h. The decoction method was performed thrice to ensure complete extraction of the active metabolites. After each boiling step, the decoction was filtered to remove the solid plant materials. The combined filtrates were concentrated under reduced pressure and subsequently lyophilized to obtain 24.6 g of the lyophilized powder (HGJ extract) for further analysis and application. The chemical metabolites of the HGJ extract were characterized using ultra-performance liquid chromatography coupled with high-resolution mass spectrometry (UPLC-HRMS). Elaborate particulars of the analytical methodology are provided in the Supplementary Material.

### 2.2 Animals and treatments

This study was approved by the Institutional Animal Care and Use Committee of Minzu University of China (Approval No. ECMUC2023009AO). Six-week-old male C57BL/6J mice (weighing 18 ± 2 g) of SPF grade were sourced from SiPeiFu (Beijing, China). The animals were maintained under standard conditions in a ventilated cage system. All procedures complied with relevant ethical regulations and adhered to the ARRIVE reporting guidelines. CLF was induced in mice by administering a diet containing 0.1% DDC. The DDC mouse model effectively mimics the key pathological features of human CLF, including abnormal serum biochemical parameter levels, liver fibrosis, cholestasis, inflammatory infiltration, and ductular reactions (Lee and Seki, 2023; Muns et al., 2024; Yao et al., 2023; Yao et al., 2024). Previous studies have shown that 2–4 weeks of 0.1% DDC feeding significantly increases the levels of markers of hepatic damage, cholestasis, and fibrosis (Baek et al., 2025; Du et al., 2025; Li Q. et al., 2025; Zhang et al., 2024). Forty mice were randomly allocated into five groups (n = 8): a normal group fed a standard chow diet, and four experimental groups–model, OCA, low-dose HGJ (HGJ-L), and high-dose HGJ (HGJ-H), which were administered a 0.1% DDC diet (SiPeiFu, Beijing, China). The HGJ-L and HGJ-H groups received daily oral gavage of 0.5 and 1.0 g/kg HGJ extract, respectively, with the 1.0 g/kg dose representing the human-equivalent dose. The OCA group received 6.5 mg/kg OCA (Aladdin, Shanghai, China) through oral gavage. Equivalent volumes of distilled water were administered to the normal and model groups. Following the 2-week investigational period, all mice were anesthetized using isoflurane, and blood samples were collected. Following euthanasia by cervical dislocation, liver and fecal specimens were collected for further analysis. The levels of serum biochemical markers were measured using samples from eight mice per group. Liver histopathological sections were prepared from three randomly selected mice per group. Samples for 16S rRNA sequencing, metabolomics, and transcriptomics analyses were obtained from six randomly selected mice per group.

### 2.3 Biochemical and histopathological analyses

Serum biochemical markers, including alanine aminotransferase (ALT), alkaline phosphatase (ALP), total bile acid (TBA), total bilirubin (TBIL), collagen type IV (Col-IV), hyaluronidase (HAase), procollagen type III (PC-III), and laminin (LN), were measured with commercial kits from Nanjing Jiancheng (Jiangsu, China; for ALT, ALP, TBA), Solarbio (Beijing, China; for TBIL), and Kete (Jiangsu, China; for Col-IV, HAase, PC-III, LN), following the manufacturers’ protocols. Liver specimens were processed for histological evaluation, which involved fixation in 10% formalin, paraffin embedding, and sectioning (5 μm). This enabled morphological evaluation of hematoxylin and eosin (H&E)-stained sections, whereas collagen deposition was assessed in sections with Masson’s trichrome and Sirius red staining.

### 2.4 16S rRNA gene sequencing

Microbial profiling of the gut microbiota using 16S rRNA gene sequencing was performed on fecal samples from the Normal, Model, and HGJ-H groups. The detailed experimental methods are provided in the Supplementary Material. Briefly, used amplification of the bacterial 16S rRNA gene V3–V4 hypervariable regions using the 338F/806R primer pair. The generated amplicons were subsequently purified and processed for paired-end sequencing using an Illumina NextSeq 2000 platform (Illumina, San Diego, United States).

### 2.5 Untargeted metabolomics analysis

Serum samples (100 μL) from the model and HGJ-H groups were processed and analyzed using untargeted metabolomics. Detailed experimental methods are provided in the Supplementary Material. Metabolites were identified using the Human Metabolome Database (HMDB), as well as the Majorbio and Metlin databases. Significantly altered metabolites were selected based on variable importance in projection (VIP) > 1 using orthogonal partial least squares discriminant analysis (OPLS-DA) models and p < 0.05 from the Student’s t-test.

### 2.6 Transcriptomics analysis

Liver transcriptomes from the model and HGJ-H groups were analyzed using RNA sequencing, with full methodological details provided in the Supplementary Material. Differentially expressed genes (DEGs) were screened for by applying thresholds of |log2FC| ≥ 1 and a false discovery rate (FDR) of <0.05. The resulting set of DEGs were further subjected to protein-protein interaction (PPI) network analysis, Gene Ontology (GO) annotation, and Kyoto Encyclopedia of Genes and Genomes (KEGG) pathway enrichment.

### 2.7 Reverse transcription-quantitative polymerase chain reaction (RT-qPCR) and Western blotting (WB)

Relative mRNA expression levels of aminolevulinic acid synthase 1 (Alas1), serine dehydratase (Sds), serine dehydratase-like (Sdsl), alpha-smooth muscle actin (α-SMA), and collagen type I (Col-I) in liver tissue were determined using RT-qPCR, using Gapdh for normalization and the 2^−ΔΔCT^ method for calculation. The corresponding primer sequences are provided in Supplementary Table S1. WB analysis was performed to quantify the protein expression of Alas1, Sds, Sdsl, α-SMA, and fibronectin (FN) in liver tissues, with Gapdh serving as the loading control. The primary antibodies included anti-Alas1 (1:500; Servicebio), anti-Sds (1:2000; ABclonal), anti-Sdsl (1:4000; Servicebio), anti-α-SMA (1:1000; Servicebio), anti-FN (1:6000; ABclonal), and anti-Gapdh (1:50000; ABclonal). Secondary antibodies (1:10,000; ABclonal) were used for the signal development.

### 2.8 Statistical analysis

Data are reported as mean ± standard deviation and were analyzed using GraphPad Prism 9 (GraphPad Software, San Diego, CA, United States). Group comparisons were performed using the Student’s t-test (two groups) or one-way analysis of variance (ANOVA) (multiple groups). Associations were assessed using Spearman’s correlation, and statistical significance was set at p < 0.05.

## 3 Results

### 3.1 HGJ ameliorated CLF in model mice

**FIGURE 1:**
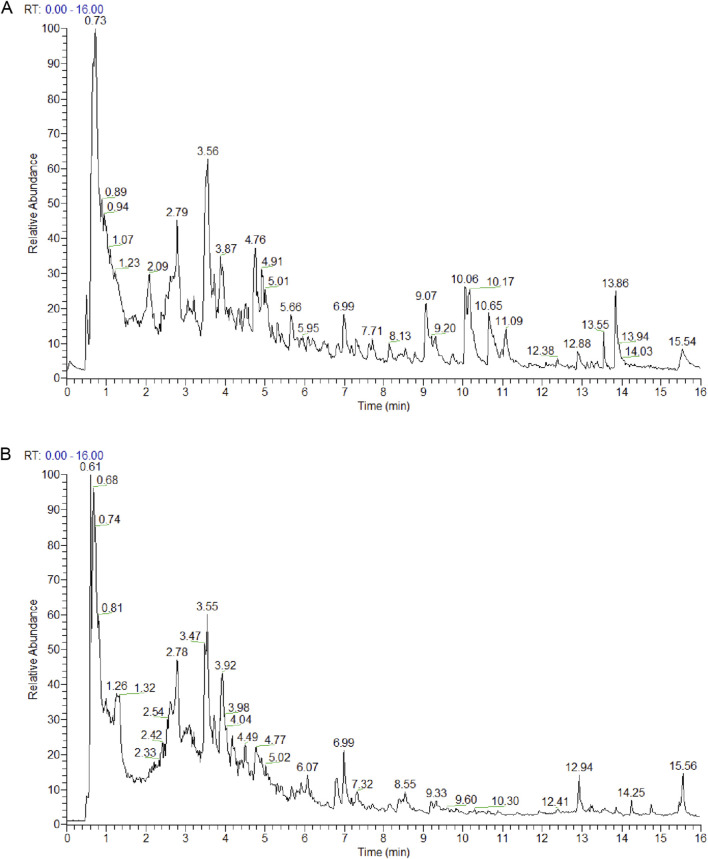
UPLC-HRMS analysis of the chemical composition of HGJ extract. TIC showing the complex mixture of metabolites of HGJ detected using UPLC-HRMS in **(A)** positive and **(B)** negative ion modes.

**FIGURE 2:**
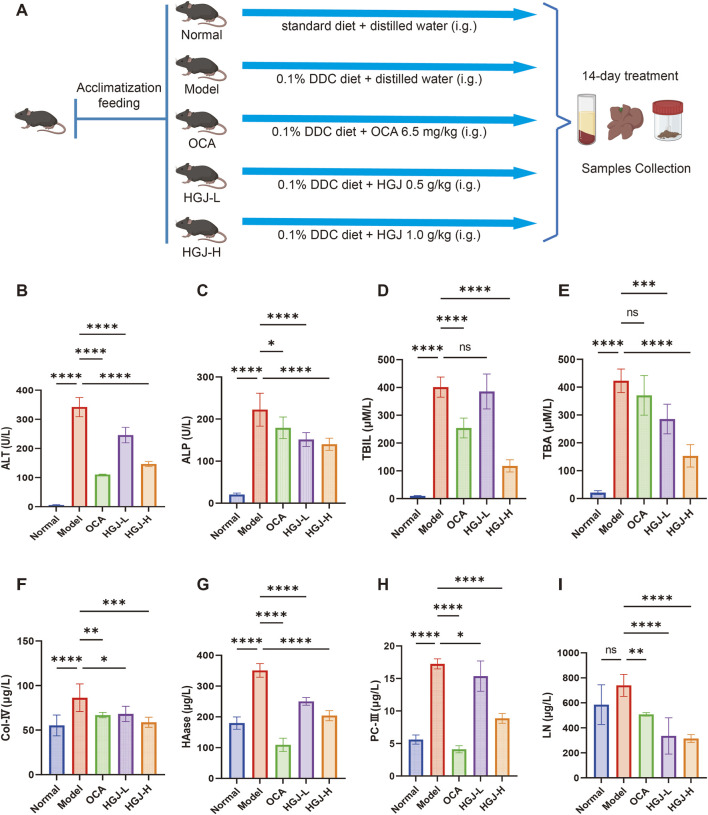
HGJ treatment reduced serum biochemical marker levels. Mice were fed 0.1% DDC to induce CLF. The HGJ-L and HGJ-H groups were administered 0.5 and 1.0 g/kg of HGJ extract, respectively, through gavage for 2 weeks. Thereafter, the mice were sacrificed, and serum samples were collected for analysis. **(A)** Workflow of the animal experiments. **(B)** Serum ALT levels. **(C)** Serum ALP levels. **(D)** Serum TBIL levels. **(E)** Serum TBA levels. **(F)** Serum Col-IV levels. **(G)** Serum HAase levels. **(H)** Serum PC-III levels. **(I)** Serum LN levels. (n = 8, mean ± SD, *p < 0.05, **p < 0.01, ***p < 0.001, ****p < 0.0001, ns: not significant; one-way ANOVA).

The HGJ extract was analyzed, and the resulting representative total ion chromatograms (TICs) are presented in Figures 1A,B. Through database comparisons, 724 metabolites were identified in the HGJ extract (Supplementary Table S2). To investigate the effects of HGJ on CLF, CLF models were generated by feeding mice a diet containing 0.1% DDC, a well-known CLF model. After 2 weeks of treatment, liver injury and fibrosis markers were measured, and histopathological analysis of the liver tissues was conducted (Figure 2A). The model group exhibited significantly higher levels of liver injury markers (ALT and ALP) and cholestatic markers (TBIL and TBA) than the normal group. Conversely, treatment with the positive control OCA significantly lowered ALT, ALP, and TBIL levels compared with those in the model group. In addition, both the HGJ-L and HGJ-H groups showed significant, dose-dependent reductions in ALT, ALP, and TBA levels relative to those of the model group, with the HGJ-H treatment also lowering TBIL levels. Notably, HGJ-H exhibited superior anti-cholestatic effects, particularly on TBA and TBIL, than those of OCA (Figures 2B–E). Similarly, the model group exhibited markedly elevated serum fibrosis marker levels (Col-IV, HAase, and PC-III) compared with those of the normal group. In contrast, all treatment groups (OCA, HGJ-L, and HGJ-H) showed significantly decreased Col-IV, HAase, PC-III, and LN levels (Figures 2F–I).

**FIGURE 3:**
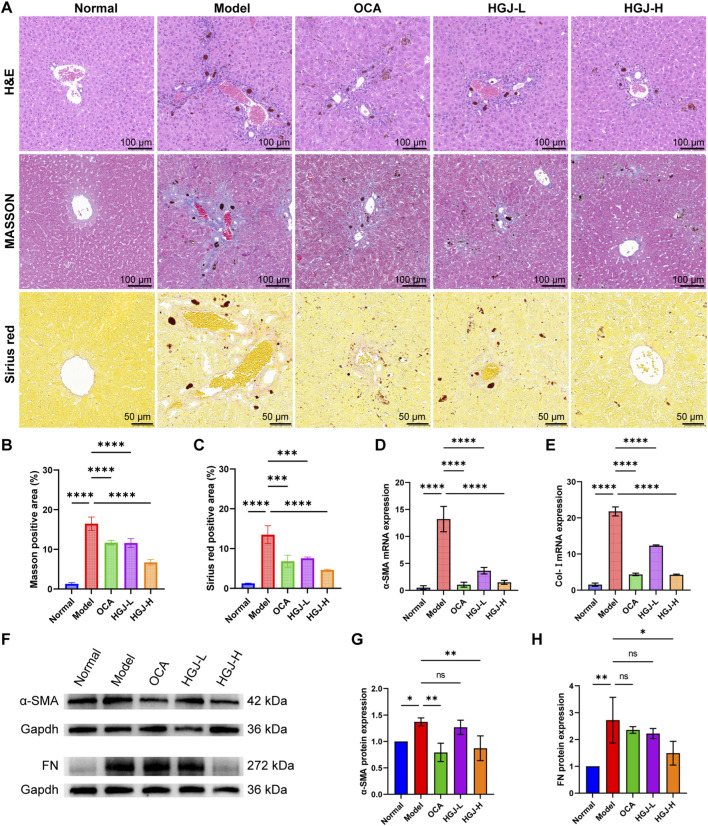
HGJ alleviated DDC-induced CLF. Mice were fed 0.1% DDC to induce CLF. The HGJ-L and HGJ-H groups were administered 0.5 and 1.0 g/kg of HGJ extract through gavage for 2 weeks. Thereafter, the mice were sacrificed, and liver samples were collected for further analysis. **(A)** Representative photomicrographs of liver sections stained with H&E, Masson’s trichrome stain, and Sirius red, respectively. **(B,C)** Quantitative analysis of the fibrotic area in Masson’s- or Sirius red-stained sections. **(D,E)** mRNA expression levels of α-SMA and Col-I. **(F)** Representative immunoblots showing protein expression of α-SMA and FN. **(G,H)** Quantification of the protein bands for α-SMA and FN. For panels **(B–E,G,H)**, n = 3 biological replicates; images in panels **(A,F)** are representative of three independent experiments. Mean ± SD, *p < 0.05, **p < 0.01, ***p < 0.001, ****p < 0.0001, ns: not significant; one-way ANOVA.

H&E staining showed normal hepatocyte arrangement and morphology in the normal group, whereas the model group exhibited severe disruption of liver tissue structure, nuclear deformation, and extensive inflammatory infiltration. The OCA, HGJ-L, and HGJ-H groups exhibited improved liver cell morphology and reduced inflammatory cell infiltration (Figure 3A). Masson’s trichrome and Sirius red staining indicated increased collagen deposition in the model group. Conversely, the groups treated with OCA, HGJ-L, and HGJ-H showed decreased fibrous deposition compared with the model group (Figure 3A). Quantitative analysis confirmed a significant increase in collagen fibers in the model group; however, a significant reduction was observed after treatment with OCA, HGJ-L, and HGJ-H (Figures 3B,C). To further evaluate the extent of liver fibrosis, the levels of fibrosis markers (α-SMA, Col-I, and FN) in liver homogenates were measured using RT-qPCR and WB. The mRNA levels of α-SMA and Col-I were markedly higher in the model group than the others, whereas both OCA and high/low doses of HGJ significantly reduced their expression (Figures 3D,E). α-SMA and FN protein expression was significantly increased in the model group. HGJ-H treatment notably decreased both α-SMA and FN protein levels, whereas OCA only reduced α-SMA expression (Figures 3F–H). These findings reveal that mice fed the DDC diet for 2 weeks developed significant cholestatic liver fibrosis, characterized by liver injury, cholestasis, and fibrosis. The positive control drugs OCA and HGJ-H effectively alleviated these pathological features. Notably, HGJ-H exhibited superior efficacy in alleviating cholestasis compared to OCA.

### 3.2 HGJ improved gut microbiota imbalances in CLF

**FIGURE 4:**
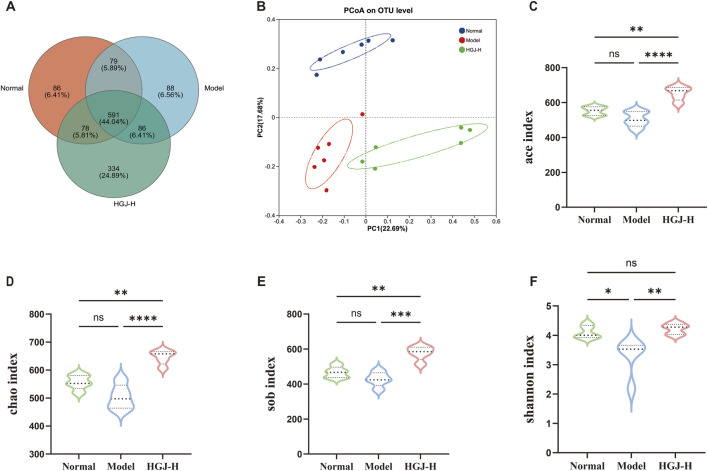
16S rRNA gene sequence analysis of fecal samples. Fecal samples from the normal, model, and HGJ-H groups were subjected to 16S rRNA gene sequencing. **(A)** Venn diagram of the OTUs across the three groups. **(B)** PCoA. **(C)** ACE index. **(D)** Chao index. **(E)** Sob index. **(F)** Shannon index. (n = 6, mean ± SD, *p < 0.05, **p < 0.01, ***p < 0.001, ****p < 0.0001, ns: not significant; one-way ANOVA).

16S rRNA gene sequencing was performed on fecal samples from the normal, model, and HGJ-H groups. The Venn diagram shows that 591 operational taxonomic units (OTUs) were shared across all groups, with 86, 88, and 334 unique OTUs in the normal, model, and HGJ-H groups, respectively (Figure 4A), indicating that HGJ intervention modulates the gut microbiota structure. Principal coordinate analysis (PCoA) showed a clear separation among the three groups, revealing a structural change in the gut microbiota (Figure 4B). Alpha-diversity analysis indicated a declining trend in the ACE, Chao1, and Sob indices in the model group relative to the normal group, accompanied by a significant decrease in the Shannon index. Following HGJ intervention, all four indices were significantly restored compared to those in the model group (Figures 4C–F). These findings collectively reveal that HGJ treatment effectively enhances both the richness and diversity of the gut microbiota.

**FIGURE 5:**
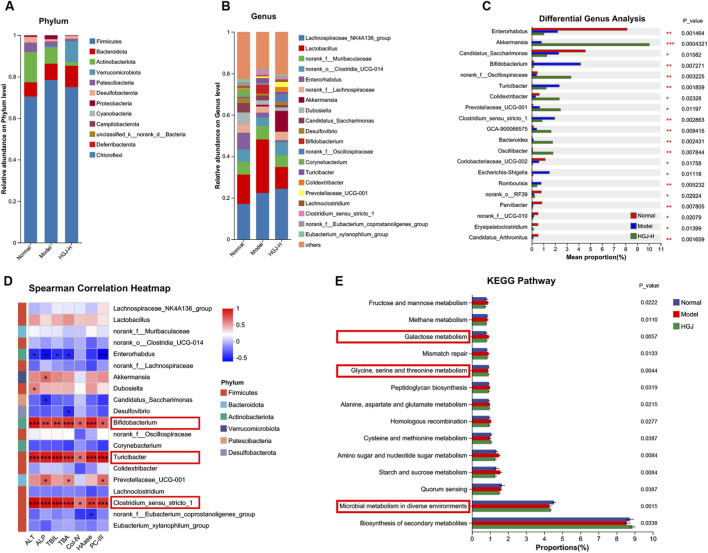
HGJ improved the gut microbiota imbalance in CLF. Fecal samples from the normal, model, and HGJ-H groups were subjected to 16S rRNA gene sequencing (n = 6). **(A)** Gut microbiota composition at the phylum level. **(B)** The top 20 bacterial genera are ranked based on relative abundance in the gut. **(C)** Significant differences in bacterial genera among the three groups (top 20). **(D)** Spearman’s correlation analysis between the gut microbiota genera and indicators of liver injury and fibrosis. The strength and direction of the correlation are indicated in different colors, as shown in the key, which corresponds to Spearman’s correlation coefficient. Statistical significance was determined after adjusting for multiple comparisons using the Benjamini–Hochberg FDR method. Asterisks denote statistically significant correlations after correction: * FDR-adjusted p < 0.05, ** FDR-adjusted p < 0.01, *** FDR-adjusted p < 0.001. **(E)** Functions of the gut microbiota (KEGG pathways).

Phylum-level analysis revealed a shift in the dominant gut microbiota following HGJ intervention. The normal and model groups were dominated by Firmicutes, Actinobacteriota, and Bacteroidota, whereas the HGJ-H group showed a notable increase in Verrucomicrobiota, forming a new dominant consortium with Firmicutes and Bacteroidota (Figure 5A). Figure 5B shows the top 20 bacterial genera based on the relative abundance of the gut microbiota. Differences in genus abundance among the three groups were analyzed, and the top 20 genera with significant differences are shown in Figure 5C. Spearman’s correlation analysis was used to assess the correlations between liver injury/fibrosis markers and the abundance of specific bacterial genera. The top 20 genera in terms of abundance are shown in Figure 5D. The analysis indicated that liver injury and fibrosis markers, including ALT, ALP, TBIL, TBA, Col-IV, HAase, and PC-III, were significantly and positively correlated with the genera *Bifidobacterium*, *Turicibacter*, and *Clostridium_sensu_stricto_1*. The Phylogenetic Investigation of Communities by Reconstruction of Unobserved States was used to predict the functions of the gut microbiota. Figure 5E shows the KEGG pathways that ranked in the top 30 based on the microbial abundance.

### 3.3 HGJ modulated the serum metabolite profiles of CLF models

**FIGURE 6:**
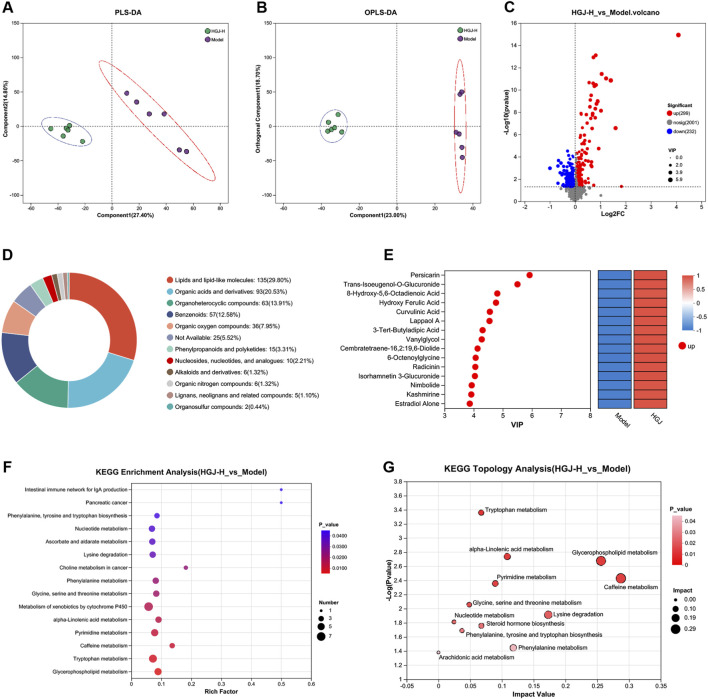
Untargeted metabolomic analysis. Serum samples from the model and HGJ-H groups (n = 6) were analyzed using untargeted metabolomic analysis. **(A)** PLS-DA. **(B)** OPLS-DA. **(C)** Volcano plot showing differential metabolites between the HGJ-H and model groups. **(D)** Annotation of differential metabolites using the HMDB. **(E)** Top 15 differential metabolites. **(F)** KEGG enrichment analysis was performed. **(G)** KEGG pathway topology analysis.

To assess the metabolic impact of HGJ, serum metabolites in the model and HGJ-H groups were profiled. Partial least squares discriminant analysis (PLS-DA) (Figure 6A) and OPLS-DA (Figure 6B) revealed clear separation between the metabolites in the two groups. A total of 531 differential metabolites were identified between the HGJ-H and model groups, of which 299 were upregulated and 232 were downregulated (Figure 6C). These metabolites were annotated against the HMDB (Figure 6D), and the top 15 metabolites, ranked by VIP scores, are displayed in Figure 6E. KEGG enrichment analysis of the 531 differential metabolites revealed 15 significantly enriched pathways (Figure 6F). Furthermore, KEGG topology analysis highlighted the key affected metabolic pathways, including caffeine metabolism and glycerophospholipid metabolism (Figure 6G).

### 3.4 Impact of HGJ on the hepatic transcriptome of CLF

**FIGURE 7:**
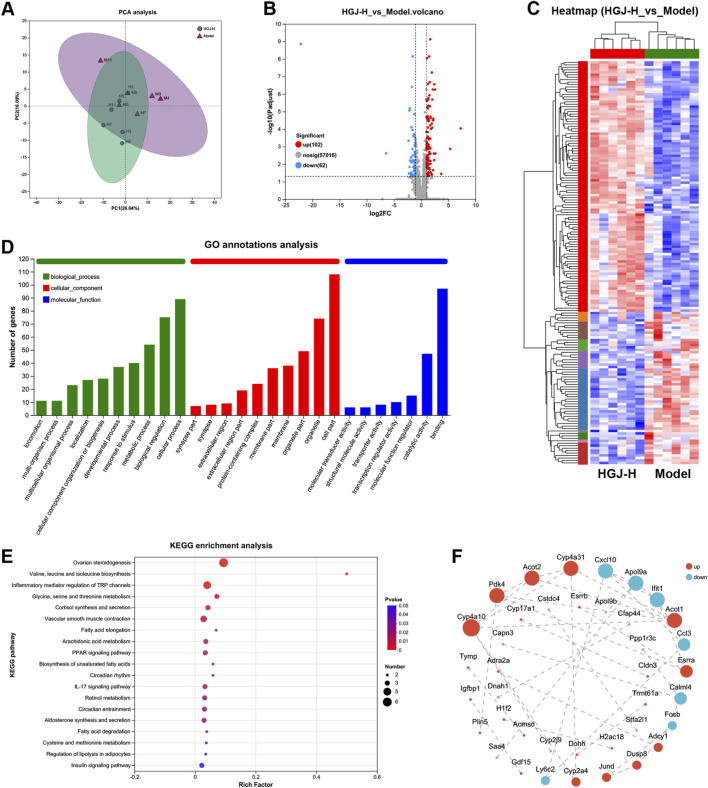
Transcriptomic analysis of the liver. Transcriptomes of liver samples from the model and HGJ-H groups (n = 6) were analyzed. **(A)** PCA of the model and HGJ-H groups. **(B)** Volcano plots showing the DEGs between the HGJ and model groups. **(C)** Cluster analysis of DEGs. **(D)** GO annotation analysis of DEGs. **(E)** KEGG pathway enrichment analysis of the DEGs. **(F)** PPI networks.

RNA sequencing of liver samples from the model and HGJ-H groups was performed to assess the HGJ-induced transcriptomic alterations. Principal component analysis (PCA) revealed a clear separation between the two groups (Figure 7A). Comparative analysis identified 164 DEGs, including 102 upregulated and 62 downregulated genes in the HGJ-H group relative to the model group (Figure 7B). Cluster analysis of the DEGs revealed a distinct clustering trend between the HGJ-H and model groups (Figure 7C). In the hierarchical clustering dendrogram, the samples from the HGJ-H group are clearly distinguishable from those of the model group (Figure 7C). GO annotation analysis of the DEGs highlighted their involvement in various biological processes, including cellular processes, biological regulation, and metabolism. In addition, the DEGs were predominantly distributed in the cell parts, particularly in the organelles. Moreover, they were associated with various molecular functions, such as binding, catalytic activity, and molecular function regulation (Figure 7D). KEGG enrichment analysis of the DEGs identified significant enrichment of multiple pathways, notably encompassing ovarian steroidogenesis, valine/leucine/isoleucine biosynthesis, inflammatory mediator regulation of TRP channels, glycine/serine/threonine metabolism, and circadian entrainment (Figure 7E). PPI analysis of the DEGs identified cytochrome P450 4a10 (Cyp4a10), pyruvate dehydrogenase kinase 4 (Pdk4), acyl-CoA thioesterase 2 (Acot2), cytochrome P450 4a31 (Cyp4a31), and chemokine ligand 10 (Cxcl10) as the core target genes (Figure 7F).

### 3.5 Integrated analysis of multi-omics data

**FIGURE 8:**
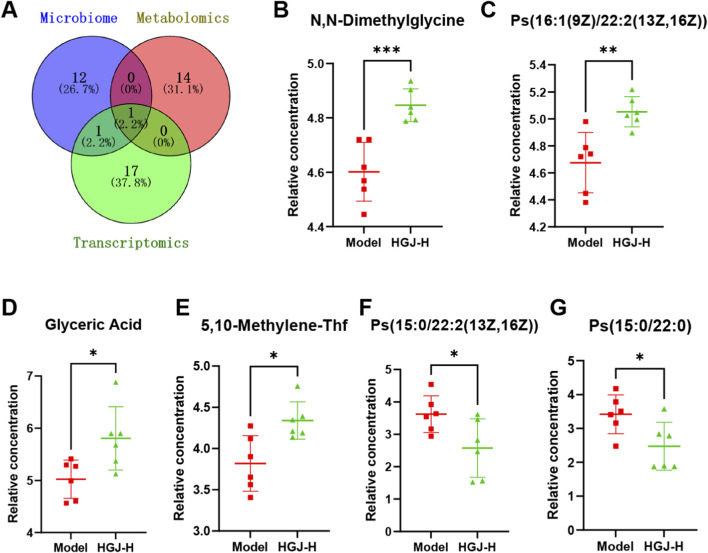
Integrated analysis of multi-omics data. **(A)** Venn diagram of the enriched KEGG pathways in the microbiome, metabolome, and transcriptome. The six differential metabolites detected in the glycine/serine/threonine metabolism pathway were as follows: **(B)** N, N-dimethylglycine, **(C)** Ps(16:1(9Z)/22:2(13Z,16Z)), **(D)** glyceric acid, **(E)** 5,10-methylene-Thf, **(F)** Ps(15:0/22:2(13Z,16Z)), and **(G)** Ps(15:0/22:0). (n = 6, mean ± SD, *p < 0.05, **p < 0.01, ***p < 0.001, ****p < 0.0001, ns: not significant; t-test for two groups).

**FIGURE 9:**
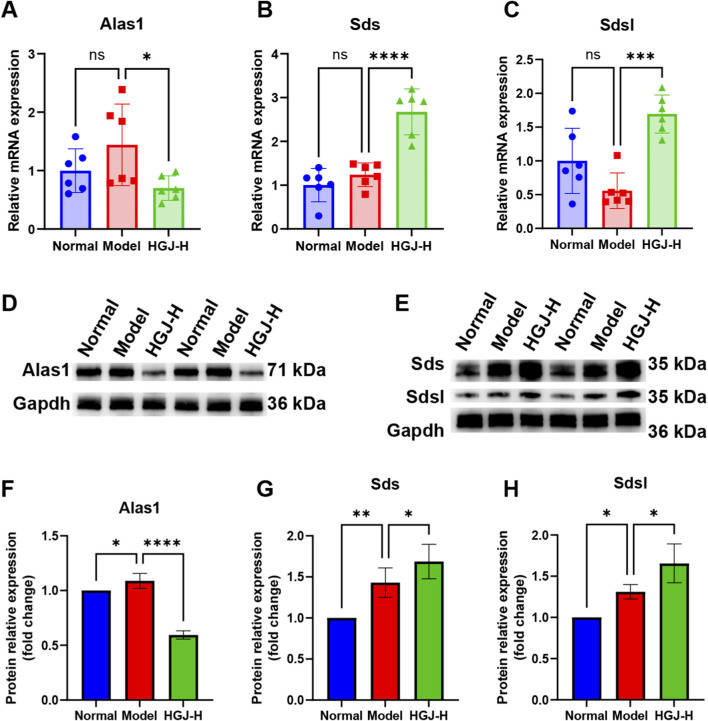
qPCR and WB experimental validation. Liver samples from the normal, model, and HGJ-H groups were analyzed using qPCR and WB. mRNA expression of **(A)** Alas1, **(B)** Sds, and **(C)** Sdsl (n = 6). Protein expression of **(D)** Alas1, **(E)** Sds, and Sdsl. Protein band quantification of **(F)** Alas1, **(G)** Sds, and **(H)** Sdsl (n = 3–4). (mean ± SD, *p < 0.05, **p < 0.01, ***p < 0.001, ****p < 0.0001, ns: not significant; one-way ANOVA).

By intersecting the KEGG pathways enriched in the microbiome, metabolome, and transcriptome data, we identified that the groups shared enrichment in the glycine/serine/threonine metabolism pathway (Figure 8A). Six differential metabolites were linked to this pathway, of which N, N-dimethylglycine, Ps(16:1(9Z)/22:2(13Z,16Z)), glyceric acid, and 5,10-methylene-Thf levels were increased, whereas Ps(15:0/22:2(13Z,16Z)) and Ps(15:0/22:0) levels were decreased (Figures 8B–G). The glycine/serine/threonine metabolism pathway also involved three DEGs: Alas1, Sds, and Sdsl. After treatment with HGJ, the mRNA expression of Alas1 in the liver tissues decreased significantly, whereas that of Sds and Sdsl increased significantly (Figures 9A–C). Corresponding to the changes in mRNA expression, the protein levels of Alas1 were notably reduced, whereas those of Sds and Sdsl were significantly increased in the liver tissues following HGJ treatment (Figures 9D–H). Collectively, integrated multi-omics analysis showed that HGJ alleviated CLF by affecting the glycine/serine/threonine metabolism pathway.

## 4 Discussion

CLF is characterized by complex pathogenesis and limited effective treatment options. Traditional Chinese medicine is beneficial for treating complex diseases. This study confirmed, using an animal model, that the classic Chinese formula HGJ can improve CLF and explored its mechanisms of action through integrated microbiome, metabolomic, and transcriptomic analyses. The results showed that HGJ enhanced the gut microbiota and reshaped the serum metabolic profile of mice with CLF. In addition integrated multi-omics analysis revealed that the glycine/serine/threonine metabolism pathway is centrally involved in the mechanisms of CLF.

In our study, PPI analysis of the DEGs identified through transcriptomics revealed that Cyp4a10, Pdk4, Acot2, Cyp4a31, and Cxcl10 are core genes regulated by HGJ. HGJ alleviates hepatocyte lipotoxicity and energy crisis by upregulating Cyp4a10/31, Pdk4, and Acot2 expression, thereby enhancing the capacity of the liver to metabolize and clear fatty acids and toxic bile acids, ultimately protecting the hepatocytes (Bjune et al., 2023; Leclercq et al., 2000). Cxcl10 is a key pro-inflammatory chemokine, and HGJ controls the hepatic inflammatory environment by downregulating Cxcl10 expression (Tokunaga et al., 2018). The synergistic action of these two mechanisms alleviates liver injury, thereby inhibiting the development of liver fibrosis.

An increase in *Bifidobacterium* abundance was observed in the DDC model, and this change was reversed by HGJ intervention. Although Bifidobacteria are generally regarded as hepatoprotective (Gavzy et al., 2023), our data revealed a positive correlation between their abundance and markers of liver injury. This seemingly paradoxical association may be interpreted in several ways. First, DDC-induced dysbiosis may selectively suppress competing microbial taxa, thereby creating an ecological niche that permits *Bifidobacterium* expansion independently of its functional role in this context (Alessandri et al., 2021). Second, the observed increase could represent a compensatory host response to hepatic injury, as suggested by elevated *Bifidobacterium* levels reported in other experimental liver disease models (Sun et al., 2025; Zheng et al., 2023).Notably, the single-timepoint sampling in our study limits causal inference, and we cannot determine whether the increase in *Bifidobacterium* levels drives pathology or is a consequence of liver damage. Thus, the proliferation of *Bifidobacterium* in our CLF model may not directly promote injury but could instead reflect a secondary adaptive or restorative reaction. Further mechanistic studies, such as longitudinal sampling, germ-free animal experiments, or targeted colonization, are required to clarify the functional contribution of *Bifidobacterium* in cholestatic fibrosis and to resolve its complex relationship with disease progression.

Integrative microbiome, metabolome, and transcriptome analyses consistently showed that the glycine/serine/threonine metabolism pathway is a major contributor to the effects of the HGJ. Serum metabolomic profiling identified six differentially abundant metabolites within this pathway: N, N-dimethylglycine, Ps(16:1(9Z)/22:2(13Z,16Z)); glyceric acid, 5,10-methylene-Thf, Ps(15:0/22:2(13Z,16Z)); and Ps(15:0/22:0). Transcriptomic data, validated using RT-qPCR and WB, revealed that HGJ downregulates Alas1 expression and upregulates Sds and Sdsl expression in the liver. N, N-dimethylglycine, a methyl donor derived from glycine, could influence epigenetic processes, such as DNA methylation (Béke et al., 2024; Lendvai et al., 2023). The phospholipids Ps(16:1(9Z)/22:2(13Z,16Z)), Ps(15:0/22:2(13Z,16Z)), and Ps(15:0/22:0) are vital membrane components that regulate membrane signaling and inflammatory responses. Glyceric acid, a product of glycolysis and glycerol metabolism, plays an important role in energy production. Notably, 5,10-methylene-Thf, a carrier of one-carbon units, supports nucleotide synthesis and methylation processes (Lakhssassi et al., 2022). Alas1 catalyzes an essential step in heme biosynthesis by combining glycine and succinyl-CoA. Its overexpression during liver injury leads to the accumulation of toxic heme precursors, increased mitochondrial oxidative stress, and collagen buildup (Balwani et al., 2020; Yasuda et al., 2023). Conversely, Sds and Sdsl degrade L-serine into pyruvate and ammonia, linking serine metabolism to the (tricarboxylic acid) TCA cycle. This process stimulates energy metabolism and increases the production of glutathione precursors, enhancing antioxidant defenses (McBride et al., 2024; Tobón-Cornejo et al., 2021).

A comprehensive assessment of microbiota, metabolites, and gene changes related to glycine/serine/threonine metabolism revealed the complex mechanisms of HGJ, which increase beneficial *Akkermansia* and decrease pathogenic *Clostridium_sensu_stricto_1*. *Akkermansia* improves intestinal barrier function, reduces endotoxin translocation, and enhances folate absorption (Cani et al., 2022; Ioannou et al., 2025). Stimultaneously, inhibiting *Clostridium_sensu_stricto_1*-mediated choline metabolism increases the serum levels of protective metabolites, such as N, N-dimethylglycine and 5,10-methylene-Thf. These metabolites subsequently enhance hepatic one-carbon metabolism and mitochondrial function. Concurrently, HGJ upregulates hepatic Sds/Sdsl expression to promote serine breakdown for energy and to supply precursors for glutathione synthesis, while downregulating Alas1 expression to reduce the diversion of glycine into toxic heme synthesis and the associated oxidative stress. These coordinated changes collectively enhance the channeling of glycine into glutathione synthesis. The combined effects thereby establish a “gut microbiota-metabolite-liver gene expression” axis that specifically improves mitochondrial function and boosts antioxidant capacity, which in turn alleviates hepatocyte injury and dampens the activation of hepatic stellate cells—key drivers of fibrogenesis—ultimately facilitating significant anti-fibrotic and hepatoprotective effects.

Although this study has revealed the novel mechanism through which HGJ improves CLF through the regulation of the glycine/serine/threonine metabolic pathway via integrated multi-omics analysis, several limitations remain to be addressed. Although the positive control drug, OCA, demonstrated favorable efficacy in the animal model, its clinical application remains underexplored, and its use in this study as an efficacy control does not constitute a clinical recommendation. Furthermore, the proposed “gut microbiota-serum metabolite-liver gene expression” axis is primarily based on correlational analyses, necessitating validation of causal relationships through experiments such as fecal microbiota transplantation or microbiota depletion. Although we have preliminarily demonstrated that this formula exerts its effects by modulating specific microbial abundances, altering serum metabolite levels, and regulating key enzyme expression, the precise regulatory network among microbes, metabolites, and genes requires further examination using methods such as gene editing and metabolite intervention. Addressing these questions will help refine the theoretical framework of the mechanism of action of the formula as well as provide important insights for developing new therapeutic strategies targeting this metabolic pathway for liver fibrosis.

## Data Availability

The raw sequencing data (16S rRNA and transcriptome) presented in the study are deposited in the NCBI repository, accession number PRJNA1433180 (available at: https://www.ncbi.nlm.nih.gov/sra/PRJNA1433180); and serum metabolomics data presented in the study are deposited in the MetaboLights repository, accession number MTBLS13999 (available at: https://www.ebi.ac.uk/metabolights/editor/MTBLS13999/descriptors).
