# Supplementary material for: Multi-omics analysis reveals the mechanism of Huaganjian in alleviating cholestatic liver fibrosis
Source: Front Pharmacol. 2026 Mar 12;17:1744312. doi: 10.3389/fphar.2026.1744312 (PMC13017810; doi:10.3389/fphar.2026.1744312)
Supplement: Supplementary file 1 [file Table1.docx]

**Supplementary information**

**List of Abbreviations**

| Acot2 | acyl-CoA thioesterase 2 |
| --- | --- |
| ALP | alkaline phosphatase |
| ALT | alanine aminotransferase |
| α-SMA | alpha-smooth muscle actin |
| Alas1 | aminolevulinic acid synthase 1 |
| CLF | cholestatic liver fibrosis |
| Col-Ⅰ | collagen type I |
| Col-Ⅳ | collagen type Ⅳ |
| Cxcl10 | chemokine ligand 10 |
| Cyp4a10 | cytochrome P450 4a10 |
| Cyp4a31 | cytochrome P450 4a31 |
| DDC | 3,5-diethoxycarbonyl-1,4-dihydrocollidine |
| DEGs | differentially expressed genes |
| ESI | electrospray ionization |
| FDR | false discovery rate |
| FN | fibronectin |
| GO | Gene Ontology |
| HAase | hyaluronidase |
| H&E | hematoxylin and eosin |
| HGJ | Huaganjian |
| HGJ-H | high-dose HGJ |
| HGJ-L | low-dose HGJ |
| HMDB | Human Metabolome Database |
| KEGG | Kyoto Encyclopedia of Genes and Genomes |
| LN | laminin |
| log FC | log2 fold change |
| OCA | obeticholic acid |
| OPLS-DA | orthogonal partial least squares discriminant analysis |
| OTUs | operational taxonomic units |
| PCA | principal component analysis |
| PC-III | procollagen type Ⅲ |
| PCoA | principal coordinate analysis |
| Pdk4 | pyruvate dehydrogenase kinase 4 |
| PLS-DA | partial least squares discriminant analysis |
| PPI | protein-protein interaction |
| RT-qPCR | reverse transcription quantitative PCR |
| Sds | serine dehydratase |
| Sdsl | serine dehydratase-like |
| TBA | total bile acid |
| TBIL | total bilirubin |
| TIC | total ion chromatograms |
| UPLC-HRMS | ultra-performance liquid chromatography coupled with high-resolution mass spectrometry |
| VIP | variable importance in the projection |
| WB | western blotting |

**Supplemental Methods**

**Analysis of HGJ Components**

Sample Preparation: A 100 μL aliquot of the concentrated HGJ extract was transferred to a 1.5 mL centrifuge tube, followed by 300 μL of an extraction solvent (methanol:acetonitrile = 1:1) containing internal standards. The mixture was vortexed for 30 s and subjected to low-temperature ultrasonic extraction for 30 min. The sample was stored at -20 °C for 30 min and centrifuged at 13000 × g (4 °C) for 15 min. The supernatant was collected for subsequent analysis.

Chromatographic Conditions: The analysis was performed using a Thermo Scientific UHPLC-Q Exactive system equipped with an ACQUITY UPLC BEH C18 column (100 mm × 2.1 mm, 1.7 μm). The mobile phase comprised (A) 2% acetonitrile in water (0.1% formic acid) and (B) acetonitrile (0.1% formic acid). The gradient elution program was as follows: 0–0.5 min (2% B), 0.5–3.5 min (2→25% B), 3.5–7.5 min (25→35% B), 7.5–11.0 min (35→50% B), 11.0–13.0 min (50→95% B), 13.0–14.4 min (95% B), 14.4–14.5 min (95→2% B), and 14.5–16.0 min (2% B). The injection volume was 3 μL, and the column temperature was maintained at 40 °C. MS detection was conducted using electrospray ionization (ESI) in both positive and negative ion modes. The scan range was 70–1050 m/z. The key parameters included: sheath gas flow rate, 50 arb; auxiliary gas flow rate, 13 arb; heater temperature, 450 °C; capillary temperature, 320 °C; spray voltage, 3500 V (positive) and -3000 V (negative); S-Lens RF level, 40 V; and normalized collision energies of 20, 40, and 60 eV.

Compound Identification: Raw data were processed using Progenesis QI v3.0 (Waters Corporation, USA) for baseline correction, peak detection, alignment, and retention time calibration, generating a data matrix with retention time, m/z, and peak intensity. The metabolites were identified by matching the MS and MS/MS spectra against the MJBIOTCM database (mass error < 10 ppm), with secondary spectral matching scores used for confirmation.

**16S rRNA Gene Sequencing Analysis**

Total microbial genomic DNA was extracted from the fecal samples of the normal, model, and HGJ-H groups using the Fast Pure Stool DNA Isolation Kit (MJYH, Shanghai, China) according to the manufacturer’s instructions. The quality and concentration of DNA were assessed using a 1.0% agarose gel electrophoresis and a NanoDrop 2000 spectrophotometer (Thermo Scientific, USA), and the samples were stored at -80 °C until further use. The hypervariable region V3-V4 of the bacterial 16S rRNA gene was amplified using the primer pairs 338F (ACTCCTACGGGAGGCAGCAG) and 806R (GGACTACHVGGGTWTCTAAT) on a T100 Thermal Cycler (BIO-RAD, USA). The PCR product was extracted from the 2% agarose gel, purified with a PCR Clean-Up Kit (YuHua, Shanghai, China) following the manufacturer’s instructions, and quantified using a Qubit 4.0 (Thermo Fisher Scientific, USA). The purified amplicons were pooled in equal molar amounts and sequenced using paired-end technology on an Illumina NextSeq 2000 platform (Illumina, San Diego, CA, USA) by Majorbio Bio-Pharm Technology Co., Ltd. (Shanghai, China), according to standard protocols.

**Untargeted metabolomics analysis**

Serum samples (100 μL) from the Model and HGJ-H groups of mice were added to a 1.5 mL centrifuge tube containing 400 μL of a 1:1 acetonitrile:methanol solution and 0.02 mg/mL internal standard (L-2-chlorophenylalanine) to extract the metabolites. The samples were vortexed for 30 s and then sonicated at 5 °C for 30 min (40 kHz). The samples were stored at -20 °C for 30 min to precipitate the proteins. Then, the samples were centrifuged for 15 min at 4°C and 13000 × g. The supernatant was removed and dried under a nitrogen atmosphere. Subsequently, the sample was re-solubilized with 100 µL of a solution (acetonitrile:water = 1:1) and extracted using ultrasonication for 5 min at 5 °C and 40 kHz, followed by centrifugation at 13000 × g and 4 °C for 10 min. Next, the supernatant was transferred to a sample vial for analysis.

Analyses were conducted using a Thermo Scientific UHPLC-Exploris 240 system coupled with a Fourier transform MS. The chromatographic conditions included an ACQUITY UPLC HSS T3 column (100 mm × 2.1 mm i.d., 1.8 μm; Waters, Milford, USA); a mobile phase consisting of (A) 95% water and 5% acetonitrile with 0.1% formic acid and (B) 47.5% acetonitrile, 47.5% isopropanol, and 5% water with 0.1% formic acid. The gradient elution program was as follows: Positive mode — 0-3 min (0→20% B), 3-4.5 min (20→35% B), 4.5-5 min (35→100% B), 5-6.3 min (100% B), and then returning to 0% B at 6.4 min. Negative mode — 0-1.5 min (0→5% B), 1.5-2 min (5→10% B), 2-4.5 min (10→30% B), 4.5-5 min (30→100% B), 5-6.3 min (100% B), with re-equilibration to 0% B at 6.4 min. The total runtime for both modes was 8 min. The injection volume was 3 μL, and the column temperature was kept at 40°C.

MS conditions: The samples were ionized using ESI, and MS data were collected in both the positive and negative ion modes. The analysis used optimized settings: Full-scan MS data were gathered from m/z 70-1050 at a resolution of 60,000 (Full MS) and 15,000 for MS² scans. ESI was conducted at spray voltages of +3,400 V (positive mode) and -3,000 V (negative mode), with the sheath and auxiliary gas flows set to 60 and 20 arbitrary units, respectively. The ion transfer tube and vaporizer temperatures were kept at 320°C and 350°C.

The metabolites were identified using the Human Metabolome (http://www.hmdb.ca/), Metlin (https://metlin.scripps.edu/), and the Majorbio databases. The selection of significantly different metabolites was based on the variable importance in the projection (VIP) scores from the orthogonal partial least squares discriminant analysis (OPLS-DA) model and p-values from the student’s t-test. Metabolites with VIP > 1 and p < 0.05 were considered significantly different.

**Transcriptomics analysis**

Total RNA was extracted from the liver tissues of normal and high-dose HGJ-treated mice using the MJZol Total RNA Kit (Majorbio, Shanghai, China). RNA quality was verified using the Nanodrop2000 (OD260/280:1.8-2.2), agarose gel electrophoresis, and Agilent 5300 analysis (RIN>6.5). Stranded mRNA libraries were prepared using the Illumina Stranded mRNA Prep Kit and sequenced on a NovaSeq XPlus platform. The workflow included mRNA enrichment with oligo(dT) beads, fragmentation (300-400 bp), cDNA synthesis, end repair, and adapter ligation. Raw data were quality controlled using FASTP, then aligned to the reference genome using HISAT2. Transcript assembly and quantification were performed using StringTie and RSEM. Differentially expressed genes (DEGs) were identified with DESeq2 (|log2 fold change (log FC)| ≥ 1, false discovery rate (FDR) < 0.05). In addition, DEGs were subjected to protein-protein interaction (PPI) analysis, Gene Ontology (GO) annotation, and Kyoto Encyclopedia of Genes and Genomes (KEGG) enrichment analysis.

**Supplemental Table 1：Primer sequences**

| Gene name | Forward/  Reverse | Sequences (5’-3’) |
| --- | --- | --- |
| α-SMA | Forward | CCCTGAAGAGCATCCGACAC |
|  | Reverse | CCAGAGTCCAGCACAATACCA |
| Col-Ⅰ | Forward | GACATGTTCAGCTTTGTGGACCTC |
|  | Reverse | GGGACCCTTAGGCCATTGTGTA |
| Alas1 | Forward | GGCGGATGACTACACGGATT |
|  | Reverse | CAGTGCCTGCTCCAGTTCTA |
| Sds | Forward | TGGTGGGAGAGATGCTGGAT |
|  | Reverse | ACAAGGGAAGTGTGGCCTTC |
| Sdsl | Forward | GCTGCATACTCGGCTCGTAA |
|  | Reverse | GTCAAACGGGGAGACGTTCA |
| Gapdh | Forward | AAATGGTGAAGGTCGGTGTGAAC |
|  | Reverse | CAACAATCTCCACTTTGCCACTG |

**Supplemental Table 2：Compounds in HGJ extract**

| **Compound** | **m/z** | **Retention time** | **Mode** |
| --- | --- | --- | --- |
| Kashmirine | 430.3309654 | 4.9122 | pos |
| Nobiletin | 403.1380513 | 10.05905 | pos |
| 2-(3,4,5-Trimethoxyphenyl)-5,6,7,8-tetramethoxy-4H-1-benzopyran-4-one | 433.1486689 | 10.65035 | pos |
| Sinensetin | 373.1273912 | 9.073033333 | pos |
| Proline betaine | 144.1016923 | 0.656933333 | pos |
| Tetramethylscutellarein | 343.116945 | 10.16663333 | pos |
| Peimine | 432.3465711 | 5.658983333 | pos |
| Pc(34:2) | 758.568456 | 14.7288 | pos |
| Citric acid | 191.0186011 | 0.68075 | neg |
| Tangeretin | 373.1276942 | 11.08543333 | pos |
| Aloenin | 411.1254802 | 3.556516667 | pos |
| Gallic acid | 169.0130052 | 1.301416667 | neg |
| Naringenin 7-rutinoside | 579.1717404 | 4.514233333 | neg |
| Hesperidin | 609.1821722 | 4.771766667 | neg |
| Albiflorin | 481.1698585 | 3.72205 | pos |
| 2-Hydroxycinnamaldehyde | 149.0595701 | 3.556516667 | pos |
| Isosinensetin | 373.1274733 | 8.12995 | pos |
| Indoline | 120.0808486 | 2.0875 | pos |
| Peimisine | 428.3154927 | 4.585633333 | pos |
| Sinapyl aldehyde | 209.0805275 | 3.556516667 | pos |
| 7-methoxycoumarin | 177.0544088 | 3.53855 | pos |
| 5-Hydroxy-3,6,7,8,3',4'-hexamethoxyflavone | 419.1330502 | 10.97596667 | pos |
| Paeoniflorigenone | 319.117073 | 3.72205 | pos |
| 6-Demethoxytangeretin | 343.1169775 | 9.309933333 | pos |
| Yibeissine | 444.3102114 | 4.038816667 | pos |
| Oxypaeoniflorin | 495.1504246 | 3.05735 | neg |
| Alisol A | 535.3633616 | 12.93798333 | neg |
| Edpetiline | 592.3837486 | 4.39665 | pos |
| 9,12,13-Todea | 329.2328366 | 8.5469 | neg |
| Solasodine | 414.3360073 | 6.179983333 | pos |
| D-(-)-Quinic acid | 191.0548344 | 0.609266667 | neg |
| Didymin | 593.1872298 | 6.073533333 | neg |
| Choline | 104.1071495 | 0.6028 | pos |
| (6E)-8-Oxogeraniol | 169.1220827 | 3.873066667 | pos |
| Puqiedinone | 414.3359563 | 6.4776 | pos |
| Glucoliquiritin | 581.1857429 | 7.02675 | pos |
| Paeoniflorin | 479.1554979 | 3.922583333 | neg |
| Genipin | 225.0760272 | 3.54625 | neg |
| Acetophenone | 121.0648058 | 3.53855 | pos |
| Hexamethylquercetagetin | 403.1380653 | 9.72895 | pos |
| Trehalose | 341.1083508 | 0.627033333 | neg |
| Hesperetin | 303.0857564 | 4.761166667 | pos |
| 3-Hydroxy-3-methylglutarate | 161.0442559 | 0.772283333 | neg |
| Benzoyloxypaeoniflorin | 599.1767758 | 5.678866667 | neg |
| Neohesperidin | 611.1962325 | 4.761166667 | pos |
| 4-Methylumbelliferone | 177.0544567 | 4.9122 | pos |
| Hymecromone methyl ether | 191.0700194 | 3.53855 | pos |
| Vicenin 2 | 595.1650687 | 3.426033333 | pos |
| 4-Hydroxy-3-methoxycinnamaldehyde | 179.0700511 | 3.9459 | pos |
| Tomatidine | 416.3516708 | 5.448583333 | pos |
| L-arginine | 175.1186555 | 0.6028 | pos |
| Vicenin II | 593.1509889 | 3.433633333 | neg |
| L-Phenylalanine | 166.0861369 | 2.0875 | pos |
| 1,2,3,6-Tetra-O-galloyl-beta-D-glucose | 787.0994832 | 3.942433333 | neg |
| Geniposide | 387.1294054 | 3.54625 | neg |
| Xanthoxyline | 197.0806373 | 3.72205 | pos |
| 2-Phenylethyl formate | 151.075215 | 3.909233333 | pos |
| 1,2,3,4,6-Pentagalloylglucose | 939.1101651 | 4.242183333 | neg |
| Hastatoside | 427.1206205 | 2.60665 | pos |
| Petilidine | 416.3514385 | 7.36075 | pos |
| Adenosine | 268.1035606 | 1.083966667 | pos |
| Genipin 1-O-beta-D-gentiobioside | 549.1821917 | 3.214133333 | neg |
| 5-O-Demethylnobiletin | 389.1226679 | 11.68655 | pos |
| 2-Pyrrolidinecarboxylic acid | 116.0706756 | 0.727933333 | pos |
| 6'-o-p-coumaroylgenipin gentiobioside | 695.218928 | 4.792716667 | neg |
| Paeonol | 167.0700086 | 7.648283333 | pos |
| Isoferulic acid | 177.0545365 | 3.2126 | pos |
| Paeonolide | 483.1465661 | 3.50245 | pos |
| Scoparone | 207.0650309 | 4.32065 | pos |
| Limocitrin | 347.0755273 | 4.78035 | pos |
| Phloroglucinol | 127.0389587 | 2.107583333 | pos |
| Naringenin | 273.0753721 | 4.50955 | pos |
| Ellagic acid | 300.9985426 | 4.039716667 | neg |
| 3-(4-Hydroxy-3-methoxyphenyl)propionic acid | 197.0805929 | 5.028516667 | pos |
| Sarracenin | 227.0909794 | 3.50245 | pos |
| D-(+)-Malic acid | 133.0127696 | 0.6626 | neg |
| 1,3,6-tri-o-galloyl-beta-d-glucose | 635.0884981 | 3.473033333 | neg |
| P-Tolualdehyde | 121.0648064 | 1.299333333 | pos |
| Rhodiocyanoside A | 260.112274 | 0.6388 | pos |
| Agarotetrol | 319.1170213 | 5.028516667 | pos |
| Scyllo-Inositol | 179.054922 | 0.609266667 | neg |
| 16-Oxoalisol A | 505.3514863 | 9.204283333 | pos |
| Isomeranzin | 261.1117114 | 5.327133333 | pos |
| Naringenin 7-rhamnoglucoside | 581.1860166 | 4.50955 | pos |
| Topotecan | 439.1971325 | 13.12036667 | pos |
| Vicenin-1 | 623.1613954 | 3.54625 | neg |
| Isoliquiritin | 419.1332409 | 4.50955 | pos |
| 5-O-Caffeoyl-4-O-Sinapoylquinic Acid | 559.1452132 | 5.194366667 | neg |
| 5,7-dimethoxycoumarin | 207.0649654 | 5.184583333 | pos |
| D-Pipecolic acid | 130.0861884 | 0.727933333 | pos |
| Berberine | 336.1224157 | 14.32168333 | pos |
| Artemetin | 389.1223676 | 10.21051667 | pos |
| Rutin | 609.1457358 | 4.059533333 | neg |
| D-(-)-Ribose | 131.0335706 | 2.22385 | neg |
| Chemanox 22 | 367.2635604 | 14.2483 | neg |
| 1-phenylpropane-1,2-dione | 166.0861024 | 1.354466667 | pos |
| Isosakuranetin-7-o-neohesperidoside | 595.2013386 | 6.073533333 | pos |
| Palmatine | 352.1536702 | 14.32168333 | pos |
| Limonin | 471.2004419 | 9.309933333 | pos |
| Tachioside | 267.0857224 | 6.9881 | pos |
| Chrysin | 287.0908751 | 6.073533333 | pos |
| 1-O-Caffeoylquinic acid | 353.0873775 | 3.173783333 | neg |
| Citropten | 207.0649575 | 5.409116667 | pos |
| Coumarin | 147.0439031 | 4.798633333 | pos |
| 3-(2-hydroxyphenyl)propanoic acid | 149.0596752 | 3.2126 | pos |
| Tianshic acid | 329.2328097 | 9.530216667 | neg |
| Beta-Glucogallin | 331.066773 | 1.221066667 | neg |
| Gulonic acid | 195.04978 | 0.591516667 | neg |
| Tyramine | 120.080879 | 1.354466667 | pos |
| L-(-)-3-Phenyllactic acid | 167.0701016 | 3.50245 | pos |
| Hesperetin 5-O-glucoside | 465.1383264 | 4.761166667 | pos |
| 5-Hydroxymethyl-2-Furaldehyde | 109.028615 | 2.107583333 | pos |
| Feruloylputrescine | 265.1542938 | 3.043166667 | pos |
| 3'-demethylnobiletin | 389.1222588 | 8.43865 | pos |
| Methyl ferulate | 209.0806826 | 3.2126 | pos |
| Bannamurpanisin | 403.1379649 | 8.787016667 | pos |
| Auraptenol | 261.1116277 | 8.787016667 | pos |
| L-isoleucine | 132.1018315 | 1.226933333 | pos |
| Gardenoside | 403.1240978 | 2.741116667 | neg |
| 2-isopropylmalic acid | 175.0599916 | 3.135183333 | neg |
| Crocin 3 | 651.2655266 | 10.63245 | neg |
| Azelaic acid | 187.0964215 | 4.917533333 | neg |
| Mellein | 179.0700167 | 5.028516667 | pos |
| Sucrose | 365.1047187 | 0.745683333 | pos |
| Andrographolide | 351.2134371 | 7.604166667 | pos |
| Cis-Aconitate | 173.0078816 | 0.68075 | neg |
| 1-O-Sinapoyl-beta-D-glucose | 385.1135711 | 3.54625 | neg |
| Crocin | 975.3704185 | 7.900066667 | neg |
| 5,7,3',4'-Tetrahydroxy-6,8-dimethoxyflavone | 347.0756524 | 4.566816667 | pos |
| Vidarabine | 268.1033918 | 0.745683333 | pos |
| Secologanin | 421.172349 | 9.160466667 | pos |
| 1,6-bis-O-galloyl-beta-D-glucose | 483.0775612 | 2.641216667 | neg |
| 6-Methylcoumarin | 161.0595509 | 3.909233333 | pos |
| L-Tyrosine | 182.0809819 | 0.995083333 | pos |
| Rhoifolin | 579.1703872 | 4.491533333 | pos |
| Trigonelline | 138.0547889 | 0.6028 | pos |
| Penduletin 4'-Methyl Ether | 359.1120692 | 11.2406 | pos |
| Naringenin-7-O-beta-D-glucoside | 435.1280937 | 4.50955 | pos |
| Isorhamnetin-3-O-gentiobioside | 621.1457014 | 4.813566667 | neg |
| 4-Methoxycinnamic acid | 179.0699587 | 6.9881 | pos |
| Gardenin a | 419.1332557 | 12.26953333 | pos |
| 3-(3-Hydroxyphenyl)propanoic acid | 167.0701706 | 4.30095 | pos |
| Isopropyl 4-Hydroxybenzoate | 181.0856233 | 3.556516667 | pos |
| Alpha-Methylene-Gamma-Butyrolactone | 99.04436067 | 1.6608 | pos |
| (E)-7-(4-hydroxy-3-methoxyphenyl)-1-phenylhept-4-en-3-one | 311.16349 | 7.712466667 | pos |
| Cinnamaldehyde | 133.0646754 | 5.028516667 | pos |
| Morin | 303.0494598 | 4.057033333 | pos |
| Corchorifatty acid F | 327.2171842 | 7.60755 | neg |
| Picrocrocin | 331.1744576 | 3.873066667 | pos |
| Chalconaringenin | 273.0752335 | 6.80105 | pos |
| Adenine | 136.0616824 | 1.083966667 | pos |
| 3-Methylbenzaldehyde | 121.0648914 | 3.2126 | pos |
| Methyl cinnamate | 163.0751495 | 3.556516667 | pos |
| Piperidine | 86.09681349 | 0.7812 | pos |
| L-Leucine | 132.1017955 | 0.7812 | pos |
| 9-Oxo-10,12-octadecadienoic acid | 295.2261423 | 8.545883333 | pos |
| (+/-)-Catechin | 289.0714631 | 3.078983333 | neg |
| 3,4-dimethylbenzoic acid | 133.0647158 | 3.72205 | pos |
| Isoscoparin | 463.1228765 | 4.3388 | pos |
| Isosakuranin | 449.1434929 | 6.073533333 | pos |
| 1,3-Dicaffeoylquinic acid | 515.1190235 | 4.70725 | neg |
| Synephrine | 168.101624 | 0.727933333 | pos |
| Eriocitrin | 595.1667906 | 4.120516667 | neg |
| Benzene-1,2,4-triol | 144.0654865 | 0.834466667 | pos |
| Homoeriodictyol | 301.0712057 | 7.317216667 | neg |
| Phenylacetonitrile | 118.0652107 | 3.53855 | pos |
| Eupatorin | 345.0962558 | 9.204283333 | pos |
| 6-hydroxy-2-aminopurine | 152.0564843 | 1.137316667 | pos |
| Cirsimaritin | 313.0713459 | 8.882066667 | neg |
| Cnidioside A | 369.1174003 | 5.104833333 | pos |
| 3-Coumaric acid | 147.0439679 | 2.58845 | pos |
| L-Tryptophan | 205.0970257 | 2.77455 | pos |
| Cyclopamine | 412.3203522 | 5.490216667 | pos |
| Lawsone methyl ether | 189.0545196 | 2.7368 | pos |
| Isoeugenitol | 207.0650748 | 2.58845 | pos |
| Ingol | 367.2085192 | 6.115533333 | pos |
| Alisol B 23-acetate | 515.3720742 | 13.85673333 | pos |
| 3,4,3'-Tri-O-methylellagic acid | 343.0452839 | 7.339516667 | neg |
| Yangonin | 276.1227168 | 3.043166667 | pos |
| Geniposidic acid | 373.1136198 | 2.443833333 | neg |
| Troxerutin | 741.2243254 | 3.640783333 | neg |
| Loganic acid | 381.1173362 | 5.949766667 | pos |
| (3R)-4'-Methoxy-2',3,7-Trihydroxyisoflavanone | 301.0711685 | 4.7499 | neg |
| 5'-S-Methyl-5'-thioadenosine | 298.0963789 | 2.7558 | pos |
| Shanzhiside | 391.1244087 | 1.89625 | neg |
| Diosmetin-7-o-rutinoside | 609.1806935 | 4.585633333 | pos |
| 2-methylpyridin-3-ol | 110.0601686 | 0.977316667 | pos |
| Paeonilactone C | 319.1169392 | 7.188483333 | pos |
| Kushenol J | 615.148399 | 4.514233333 | neg |
| 3,4-di-o-caffeoylquinic acid | 515.118539 | 4.4297 | neg |
| 4-tert-butylcatechol | 167.1065735 | 2.532733333 | pos |
| 1,4-naphthoquinone | 159.0438498 | 3.53855 | pos |
| 4-Ethylphenol | 123.0804579 | 3.909233333 | pos |
| Paeonoside | 327.1081775 | 3.527983333 | neg |
| Epinepetalactone | 167.1065259 | 2.7558 | pos |
| Amaroswerin | 603.1676742 | 4.50955 | pos |
| Protocatechuic acid | 153.0180307 | 2.365433333 | neg |
| Alisol b | 473.3620323 | 12.92023333 | pos |
| Ibuprofen | 207.1377165 | 3.628583333 | pos |
| Salvigenin | 329.1016352 | 11.86611667 | pos |
| Perillene | 151.1115571 | 3.835166667 | pos |
| Caffeoyl quinic acid | 353.0873429 | 2.641216667 | neg |
| 3-Hydroxypicolinic acid | 140.0340951 | 0.7812 | pos |
| Demethylwedelolactone | 299.0191957 | 5.833216667 | neg |
| Procyanidin B2 | 577.1349876 | 2.841233333 | neg |
| Tetralin | 133.1010476 | 13.85673333 | pos |
| 3-O-Feruloylquinic acid | 367.1029946 | 3.779316667 | neg |
| Pisatin | 315.0856402 | 8.89645 | pos |
| Furfural | 97.02873499 | 0.887883333 | pos |
| 3-Hydroxybenzaldehyde | 105.0337536 | 3.72205 | pos |
| Bicyclo[4.2.0]octa-1,3,5-triene | 105.0700978 | 3.909233333 | pos |
| Yibeinoside A | 576.3886077 | 5.308233333 | pos |
| Confertifoline | 235.1687612 | 6.950266667 | pos |
| 2-methylcitric acid | 205.0342717 | 0.627033333 | neg |
| 2(5H)-Furanone | 85.02886927 | 0.887883333 | pos |
| 2-Phenylacetamide | 136.0755099 | 1.048416667 | pos |
| (R)-(+)-2-Pyrrolidone-5-carboxylic acid | 130.0498213 | 0.995083333 | pos |
| Xanthatin | 247.1324424 | 5.8037 | pos |
| Pyrogallol | 127.0390018 | 2.62525 | pos |
| Ferulic acid | 193.0495358 | 4.262366667 | neg |
| Anhydrobelachinal | 469.3307816 | 12.44926667 | pos |
| Quercetin tetramethyl (5,7,3',4') ether | 359.111716 | 7.53755 | pos |
| 5-Hydroxycoumarin | 163.0388569 | 3.079633333 | pos |
| Tryptophol | 144.0807296 | 3.3281 | pos |
| Harman | 183.0915024 | 3.964283333 | pos |
| Kaempferol 7-O-neohesperidoside | 595.1650875 | 4.149033333 | pos |
| Lawsone | 175.0389124 | 2.60665 | pos |
| Clematine | 645.1589354 | 4.771766667 | neg |
| 4-Hydroxy-2-methylacetophenone | 151.0751116 | 6.9881 | pos |
| Gallic acid 3-O-(6a(2)-O-galloyl)-I(2)-D-glucopyranoside | 483.0776767 | 2.243766667 | neg |
| Lapachol | 243.1012054 | 5.327133333 | pos |
| Multinoside A | 611.1599115 | 4.07535 | pos |
| Acetyl-11-keto-beta-boswellic acid | 513.3568952 | 13.34491667 | pos |
| Ethylbenzene | 107.0857148 | 3.909233333 | pos |
| Eremophila-1(10),8,11-triene | 203.1791558 | 6.864583333 | pos |
| Kankanoside A | 691.3178936 | 2.76235 | neg |
| 4,5-Di-O-caffeoylquinic acid methyl ester | 529.1346856 | 5.32505 | neg |
| 8-Methoxycirsilineol | 375.1065842 | 10.1886 | pos |
| 2',6'-Dihydroxy-4'-methoxyacetophenone | 183.0651003 | 4.528816667 | pos |
| Methyl 4-hydroxyphenylacetate | 211.0602516 | 2.583133333 | neg |
| Alisol C | 487.3409674 | 9.204283333 | pos |
| Luteolin 6-c-glucoside 8-c-arabinoside | 609.1456403 | 3.214133333 | neg |
| 6-O-galloyl-beta-D-glucose | 331.066685 | 0.735166667 | neg |
| Sinapyl alcohol | 193.0857844 | 3.466816667 | pos |
| Atractyloside A | 447.2232783 | 6.183966667 | neg |
| Acerinol | 487.3409268 | 10.29693333 | pos |
| Catechin | 291.0859044 | 3.061566667 | pos |
| Guvacine | 128.0705696 | 0.763433333 | pos |
| Apiopaeonoside | 459.1506561 | 3.565283333 | neg |
| Galloyl paeoniflorin | 650.2069942 | 4.39665 | pos |
| Etiocholanolone | 311.2011629 | 13.57806667 | neg |
| Coptisine | 320.0911681 | 13.94126667 | pos |
| Kaempferol-3-O-rutinoside | 593.1510169 | 4.140983333 | neg |
| Octadec-9-ene-1,18-dioic-acid | 311.2223702 | 12.51625 | neg |
| 4-p-Coumaroylquinic acid | 337.0926611 | 3.509883333 | neg |
| Dihydrocaffeic acid | 165.0544254 | 1.048416667 | pos |
| Nicotinic acid | 124.0392843 | 0.745683333 | pos |
| Hydroxytyrosol | 119.0491655 | 0.727933333 | pos |
| Gorlic acid | 279.231392 | 14.2534 | pos |
| 4-Cresol | 109.0649545 | 3.909233333 | pos |
| D-ribonolactone | 147.0285375 | 1.101833333 | neg |
| Farrerol | 301.1064564 | 5.028516667 | pos |
| Arjungenin | 503.33743 | 9.19675 | neg |
| Xylitol | 117.0547437 | 0.887883333 | pos |
| 2-Pyrrolidinone | 86.06049954 | 2.794183333 | pos |
| 3-Ethyltoluene | 121.1012253 | 4.168133333 | pos |
| Deacetyl asperulosidic acid methyl ester | 369.1174462 | 5.409116667 | pos |
| Coumaroyl quinic acid | 339.1068309 | 4.9122 | pos |
| Jervine | 426.299824 | 4.626116667 | pos |
| Alpha,4-Dimethylstyrene | 133.1011302 | 4.130566667 | pos |
| Dg(18:1/18:3) | 617.5132309 | 14.0739 | pos |
| Isoalantolactone | 233.1532309 | 6.607316667 | pos |
| Eupatilin | 343.0817087 | 9.218916667 | neg |
| 4-(dimethylamino)benzaldehyde | 191.1177233 | 1.892816667 | pos |
| Quercetin 3-galactoside | 463.0877886 | 4.201133333 | neg |
| Quercetin 3-methyl ether | 317.0650421 | 4.818516667 | pos |
| Narcissin | 625.1757557 | 4.663933333 | pos |
| Gardenin B | 359.1119631 | 8.107866667 | pos |
| Demethylnobiletin | 389.122319 | 7.973516667 | pos |
| Caffeic acid | 179.0338704 | 3.332266667 | neg |
| 4-Hydroxyquinoline | 146.0599282 | 2.77455 | pos |
| Bayogenin | 489.3571688 | 11.39503333 | pos |
| Vanillic acid | 167.0337563 | 2.543383333 | neg |
| 5-Hydroxyindole-3-acetic acid | 174.054806 | 3.9272 | pos |
| Kainic acid | 196.0967643 | 2.383216667 | pos |
| Juglone | 207.0649607 | 3.592383333 | pos |
| Loliolide | 197.1170911 | 4.626116667 | pos |
| 2,6-dimethoxybenzoic acid | 183.0649965 | 4.761166667 | pos |
| 2-Coumaroylquinic acid | 397.1136603 | 3.8 | neg |
| Lycoperodine 1 | 217.0969616 | 3.3473 | pos |
| 8-Epideoxyloganic acid | 359.134538 | 1.833766667 | neg |
| Erucamide | 338.3410206 | 14.45741667 | pos |
| 4-Hydroxybenzaldehyde | 105.0336645 | 6.9881 | pos |
| Narcissoside | 623.1614467 | 4.4297 | neg |
| O-Cymol | 135.1167474 | 4.11245 | pos |
| Shanzhiside methyl ester | 407.1569767 | 7.448733333 | pos |
| Kaempferol 3,7,4'-trimethyl ether | 329.1013649 | 6.28795 | pos |
| Benzofuran | 119.0492621 | 2.58845 | pos |
| Curcumenol | 235.1687703 | 7.669716667 | pos |
| Jasminoside B | 364.1960154 | 2.7558 | pos |
| Isosakuranetin | 285.0763273 | 10.16021667 | neg |
| Linolenyl alcohol | 282.2787005 | 13.79356667 | pos |
| Androsin | 327.1081953 | 3.922583333 | neg |
| 4-(beta-D-glucosyloxy)-3-hydroxy-benzoic acid | 315.071877 | 1.2793 | neg |
| Licochalcone B | 285.076307 | 6.073533333 | neg |
| Rosiridoside B | 463.2183666 | 4.66455 | neg |
| Santene | 123.1168076 | 3.835166667 | pos |
| Gamma-Aminobutyric Acid | 104.0708564 | 2.794183333 | pos |
| Chrysosplenetin B | 373.0922842 | 10.18288333 | neg |
| Alpha-pinene oxide | 135.1166921 | 5.067283333 | pos |
| 2-Ethyltoluene | 121.1011776 | 5.028516667 | pos |
| Quercetin | 301.034834 | 5.811366667 | neg |
| 4-Methyl-6,7-dihydroxycoumarin | 193.0494953 | 2.58845 | pos |
| N-methylflindersine | 274.1433188 | 3.72205 | pos |
| Dopamine | 118.0651642 | 5.028516667 | pos |
| Korseveriline | 432.3472374 | 6.822516667 | pos |
| Methyl-4-O-caffeoylquinate | 367.1029691 | 3.234933333 | neg |
| Dehydrolindestrenolide | 229.1219343 | 5.8037 | pos |
| D-sorbitol | 181.0705027 | 0.591516667 | neg |
| Sinapic acid | 223.0603359 | 4.262366667 | neg |
| Phenylethyl beta-d-glucopyranoside | 285.1304357 | 4.645583333 | pos |
| 5,7-dihydroxy-4-methylcoumarin | 175.0388926 | 4.32065 | pos |
| Isoscopoletin | 193.0494619 | 4.2427 | pos |
| Asebotin | 468.187279 | 13.18803333 | pos |
| Glutaric acid | 115.0391206 | 1.6608 | pos |
| D-Cathine | 134.0964065 | 2.401816667 | pos |
| Fastigenin | 343.0816668 | 9.8679 | neg |
| Amantadine | 152.1431531 | 15.16026667 | pos |
| 4-allylanisole | 149.0959806 | 3.17455 | pos |
| Cratoxylone | 487.1966702 | 8.458133333 | neg |
| Phytosphingosine | 318.2997082 | 10.91115 | pos |
| Epinephrine | 148.0755451 | 1.101766667 | pos |
| Sebacic acid | 201.1121203 | 5.92095 | neg |
| Linderene | 231.1375747 | 5.887133333 | pos |
| Santin | 345.0963146 | 9.706683333 | pos |
| Methyl caffeate | 387.1082117 | 4.201133333 | neg |
| Dehydrotrametenolic acid | 455.3514507 | 12.92023333 | pos |
| Citraconic acid | 113.0234689 | 1.226933333 | pos |
| Schizonepetoside E | 347.1707346 | 3.332266667 | neg |
| Isorhamnetin | 317.0651754 | 4.4355 | pos |
| Oleamide | 282.2787831 | 13.3223 | pos |
| 3'-Methoxypuerarin | 447.1275383 | 4.761166667 | pos |
| 3-(2-(methylamino)ethyl)-1h-indol-5-ol | 191.1177779 | 2.401816667 | pos |
| 2-Hydroxycinnamic acid | 163.0387856 | 3.961066667 | neg |
| Dihydroactinidiolide | 181.121993 | 8.284766667 | pos |
| Alisol G | 455.351398 | 13.5896 | pos |
| 4-Isopropylbenzaldehyde | 149.0959504 | 5.104833333 | pos |
| Salicylaldehyde | 105.0337004 | 5.028516667 | pos |
| 3-o-acetyl-16alpha-hydroxydehydrotrametenolic acid | 513.3550835 | 12.6305 | pos |
| 9(s)-hode | 295.2271604 | 13.15816667 | neg |
| Anethole | 149.0958707 | 3.628583333 | pos |
| Curcumin | 413.1236623 | 7.697433333 | neg |
| Sesamoside | 401.1086535 | 3.698783333 | neg |
| Apigenin | 269.045184 | 6.787066667 | neg |
| Effusanin A | 349.1978748 | 7.733183333 | pos |
| Alantolactone | 233.1532184 | 6.054166667 | pos |
| Kojic acid | 143.0338096 | 2.663416667 | pos |
| Germacrone | 219.1740031 | 5.638066667 | pos |
| (-)-Carvone | 151.1116075 | 3.136516667 | pos |
| Cnidilide | 195.1376884 | 8.545883333 | pos |
| O-Cresol | 109.0650057 | 2.986366667 | pos |
| Crocin II | 813.3180013 | 8.949416667 | neg |
| Obacunone | 455.2052734 | 5.595766667 | pos |
| 1,3-Dimethoxybenzene | 103.0545007 | 2.0875 | pos |
| Isorhoifolin | 577.1559301 | 4.4925 | neg |
| Indole | 118.0651122 | 6.9881 | pos |
| Isopeonol | 165.0544486 | 3.49155 | neg |
| 4-Hydroxycinnamyl aldehyde | 207.0653115 | 3.54625 | neg |
| Undecanedioic acid | 215.1278482 | 7.250466667 | neg |
| Phenylacetic acid | 119.0491681 | 4.798633333 | pos |
| Nomilin | 515.2269172 | 5.203716667 | pos |
| Riboflavin | 394.1757482 | 13.02988333 | pos |
| 4-hydroxyphenylacetic acid | 151.0386809 | 4.813566667 | neg |
| Gomphrenol | 313.0349209 | 7.339516667 | neg |
| Styrene | 105.0701323 | 2.58845 | pos |
| Salipurposid | 433.1136896 | 4.140983333 | neg |
| 14-Deoxyandrographolide | 317.2088005 | 13.25543333 | pos |
| (2R)-and (2S)-naringenin-7-O-beta-D-gentiobioside | 577.1558399 | 5.390833333 | neg |
| Leucanthoside | 461.1087852 | 4.34575 | neg |
| 3-(2-Hydroxy-4-methoxy-phenyl)-acrylic acid | 195.0649725 | 3.556516667 | pos |
| Alpha-Lapachone | 243.1011398 | 8.787016667 | pos |
| (-)-Abscisic acid | 263.1284404 | 5.790133333 | neg |
| Isoguanosine | 284.0985005 | 1.137316667 | pos |
| Artemisinic acid | 235.1687779 | 9.182433333 | pos |
| Nardosinone | 251.1638335 | 4.3388 | pos |
| Norcimifugin | 293.1013888 | 6.436133333 | pos |
| Scytalone | 195.0651573 | 2.401816667 | pos |
| 3-hydroxycoumarin | 163.038746 | 4.85605 | pos |
| Cinnamic acid | 131.0490565 | 6.436133333 | pos |
| Hordenine | 166.122466 | 1.448316667 | pos |
| Atractylenolide iii | 249.1480725 | 5.887133333 | pos |
| Ganhuangenin | 347.0754315 | 7.36075 | pos |
| Butyrophenone | 149.0960539 | 2.514766667 | pos |
| Gypsogenin | 471.3465806 | 12.81138333 | pos |
| Iridin | 523.1437066 | 5.907416667 | pos |
| Picolinic acid | 124.0393769 | 2.206233333 | pos |
| 4-methyl-5-thiazoleethanol | 144.0477135 | 1.737666667 | pos |
| Sphingosine | 282.2786364 | 14.23038333 | pos |
| Cytosine | 112.0506196 | 0.727933333 | pos |
| Helicid | 283.0819183 | 3.719666667 | neg |
| Curcumol | 219.173932 | 7.426566667 | pos |
| Isorhamnetin-3-O-nehesperidine | 623.1616335 | 4.66455 | neg |
| 3-hydroxy-3-methoxycarbonylpentanedioic acid | 205.0344266 | 1.772216667 | neg |
| Jaceosidin | 331.0805026 | 7.36075 | pos |
| Benzyl benzoate | 213.0907834 | 2.62525 | pos |
| 17-Hydroxylinolenic acid | 295.226405 | 12.38146667 | pos |
| 4-hydroxybenzoic acid | 139.0388935 | 3.061566667 | pos |
| Isomucronulatol 7-O-glucoside | 465.1725368 | 5.286883333 | pos |
| 2-Ethylphenol | 123.0804934 | 3.061566667 | pos |
| 1-phenyl-2-butanone | 149.0960067 | 2.7558 | pos |
| L-Homotyrosine | 196.0967096 | 2.6818 | pos |
| Hispidulin | 299.0555579 | 7.029783333 | neg |
| Irigenin | 361.0911482 | 6.200983333 | pos |
| Triptophenolide | 311.1680687 | 15.47388333 | neg |
| 3-o-acetyl-16alpha-hydroxytrametenolic acid | 515.3727499 | 13.27743333 | pos |
| Limonene oxide | 153.1271949 | 4.020166667 | pos |
| (R)-(+)-Pulegone | 153.1271818 | 4.663933333 | pos |
| Bakkenolide a | 235.1689507 | 4.566816667 | pos |
| Methylhydroquinone | 125.0597497 | 3.289116667 | pos |
| Iridotectoral A | 469.3307416 | 13.5896 | pos |
| L-Arabinose | 149.044123 | 0.627033333 | neg |
| Inosine | 267.071675 | 0.609266667 | neg |
| (2R)-5,7-dihydroxy-2-(4-hydroxyphenyl)-6-methoxy-2,3-dihydrochromen-4-one | 301.0712539 | 5.98595 | neg |
| Beta-Thujaplicin | 165.0909737 | 2.401816667 | pos |
| Boschnialactone | 155.1064808 | 4.2427 | pos |
| Dihydrocucurbitacin F | 519.3322108 | 9.777566667 | neg |
| Hexose | 215.0317194 | 0.591516667 | neg |
| (S)-(-)-Perillyl alcohol | 135.1167514 | 4.663933333 | pos |
| Glabrolide | 469.3304572 | 10.29693333 | pos |
| Pterosin Z | 233.1533127 | 4.32065 | pos |
| Scutellarein | 285.039966 | 7.008033333 | neg |
| 16alpha-Hydroxydehydrotrametenolic acid | 471.3458956 | 10.86673333 | pos |
| Retusin | 381.0940576 | 11.50638333 | pos |
| Salidroside | 301.1306189 | 9.684783333 | pos |
| 11-Keto-beta-boswellic acid | 471.3466551 | 13.3223 | pos |
| 4-hydroxy-3-methylbenzoic acid | 151.0387253 | 3.980566667 | neg |
| 5,7,4'-trimethoxyflavone | 313.1063039 | 9.905166667 | pos |
| Atractylenolide II | 233.153164 | 8.985866667 | pos |
| Suberic acid | 173.080743 | 4.140983333 | neg |
| Britannilactone | 267.1586006 | 6.457166667 | pos |
| Centaureidin | 361.0911065 | 5.907416667 | pos |
| Echinocystic acid | 437.3409651 | 12.94088333 | pos |
| Aloe-emodin | 271.0596088 | 6.80105 | pos |
| Fisetin | 287.0546049 | 4.357533333 | pos |
| Bisacumol | 219.1741918 | 12.09075 | pos |
| 3-hydroxy-1-(4-hydroxyphenyl)propan-1-one | 165.0544402 | 4.201133333 | neg |
| 4-Hydroxypyridine | 96.04467496 | 0.727933333 | pos |
| Farnesol | 245.1856082 | 3.50245 | pos |
| O-Methylacetophenone | 135.0803433 | 3.5743 | pos |
| Cis-4-heptenal | 95.08586039 | 3.909233333 | pos |
| [6]-Shogaol | 277.1781046 | 10.5165 | pos |
| N-propyl cinnamate | 208.1330707 | 3.308966667 | pos |
| Alpha-Linolenic Acid | 279.2314975 | 13.16545 | pos |
| Octadecenedioic acid | 277.2158824 | 12.85698333 | pos |
| Homoveratric acid | 195.0652229 | 3.49155 | neg |
| Tuberonic acid | 225.1123538 | 6.38465 | neg |
| Zedoarondiol | 253.1794212 | 5.184583333 | pos |
| Madecassic acid | 469.3304075 | 9.204283333 | pos |
| Isophorone | 139.1115845 | 3.835166667 | pos |
| Traumatic acid | 227.1279963 | 7.9901 | neg |
| Lucialdehyde B | 453.3357925 | 12.81138333 | pos |
| P-Coumaric acid | 165.054441 | 4.798633333 | pos |
| 17-hydroxyisolathyrol | 351.2135216 | 9.419216667 | pos |
| Ilicic acid | 235.1688782 | 5.469466667 | pos |
| 3,5-dimethylphenol | 123.0804043 | 5.028516667 | pos |
| P-Mentha-1,3,8-triene | 135.116707 | 5.99135 | pos |
| Phenol | 112.0758295 | 4.149033333 | pos |
| Ethyl gallate | 243.050399 | 2.243766667 | neg |
| Cantharidic acid | 213.0759145 | 3.15585 | neg |
| Zeatin | 220.1179674 | 2.383216667 | pos |
| (R)-ar-Turmerone | 217.15838 | 5.595766667 | pos |
| 3,5-Dimethoxy-4-hydroxybenzaldehyde | 181.0494922 | 4.557716667 | neg |
| 7-Methylcoumarin | 161.0594257 | 6.9881 | pos |
| Diosmetin | 301.0700456 | 7.02675 | pos |
| Aurantio-obtusin | 329.0661818 | 7.362066667 | neg |
| Vanillin | 153.0543982 | 4.798633333 | pos |
| (-)-Salsolinol | 162.0911306 | 5.308233333 | pos |
| 3-o-caffeoylquinic acid methyl ester | 367.1028144 | 5.3031 | neg |
| (+)-alpha-Curcumene | 203.1791596 | 10.31935 | pos |
| Alpha-Methylstyrene | 119.0856031 | 3.628583333 | pos |
| Corymbosin | 357.0974492 | 8.1025 | neg |
| 3',4',5,7-Tetramethoxyflavone | 343.1168933 | 8.743983333 | pos |
| Kaempferol | 287.0546215 | 4.50955 | pos |
| 4',7-di-o-methylnaringenin | 301.1063653 | 7.188483333 | pos |
| Dehydroalisol B 23-acetate | 495.3449782 | 13.5896 | pos |
| Costunolide | 233.1531745 | 7.9509 | pos |
| Ectocarpene | 149.132283 | 8.545883333 | pos |
| Oridonin | 329.1743102 | 5.2463 | pos |
| Parthenolide | 249.1480859 | 4.818516667 | pos |
| Nona-2,6-dienal | 139.1116039 | 4.168133333 | pos |
| Asiatic acid | 533.3478226 | 12.62825 | neg |
| Arglabin | 247.1325895 | 4.454566667 | pos |
| Beta-lapachone | 243.1011048 | 7.317183333 | pos |
| Tropic acid | 131.0491294 | 4.000966667 | pos |
| Benzyl alcohol | 91.05462986 | 3.556516667 | pos |
| N-Methylanthranilic Acid | 152.0704884 | 3.09885 | pos |
| 6-Hydroxynicotinic acid | 138.0182813 | 0.75385 | neg |
| D-Galactaric acid | 209.0291806 | 0.609266667 | neg |
| Laricitrin | 331.0454576 | 5.855333333 | neg |
| Cantharidin | 197.0805674 | 7.188483333 | pos |
| Senkyunolide F | 239.1272954 | 4.626116667 | pos |
| Benzyl butyrate | 179.1065005 | 4.626116667 | pos |
| 2-aminoacetophenone | 136.0756104 | 4.39665 | pos |
| Aucubin | 345.1186582 | 1.005516667 | neg |
| 4-Aminobenzoic acid | 138.0549012 | 3.023466667 | pos |
| Carabrol | 251.1636691 | 8.963283333 | pos |
| 9(S)-HOTrE | 295.2263695 | 12.74363333 | pos |
| 3-hydroxy-p-cymene | 151.1115687 | 5.448583333 | pos |
| 1-naphthylamine | 144.0806195 | 9.706683333 | pos |
| Jaceidin | 359.0768 | 7.429083333 | neg |
| Curdione | 237.1845792 | 11.2406 | pos |
| Casticin | 375.1067236 | 7.928333333 | pos |
| Ursolic acid | 455.3525248 | 13.88946667 | neg |
| 2-formamidobenzoic acid | 164.0340775 | 2.4646 | neg |
| Carveol | 135.1166939 | 3.628583333 | pos |
| Homovanillic acid | 183.0650486 | 4.168133333 | pos |
| Caryophyllene oxide | 221.1895793 | 8.743983333 | pos |
| Epoxylathyrol | 395.2044348 | 7.60755 | neg |
| Alpha-Corocalene | 201.1634634 | 7.294983333 | pos |
| Phenylacetone | 117.069968 | 3.52065 | pos |
| Skullcapflavone ii | 375.1067739 | 10.88876667 | pos |
| Gamma-glu-leu | 241.1186886 | 3.740183333 | neg |
| Toluate | 137.0595892 | 3.964283333 | pos |
| Melibiose | 323.0978608 | 8.168833333 | neg |
| Ganolucidic acid B | 501.3216845 | 9.822433333 | neg |
| Methylgingerol | 309.2035864 | 9.375266667 | pos |
| (2R)-Pterosin B | 219.1376051 | 5.8037 | pos |
| Ligustilide | 191.1064327 | 5.409116667 | pos |
| Vulgarin | 263.1284363 | 5.3031 | neg |
| L-Glutamic acid | 146.0444384 | 0.591516667 | neg |
| 13-OxoODE | 295.2263984 | 13.34491667 | pos |
| Piperonal | 151.0388332 | 5.388733333 | pos |
| Luteolin | 285.0400423 | 5.790133333 | neg |
| Momilactone B | 331.1873528 | 9.9502 | pos |
| Taxifolin | 303.0505545 | 5.346016667 | neg |
| Uridine | 243.0615278 | 1.024766667 | neg |
| Methyl 4-hydroxybenzoate | 153.0543836 | 3.6465 | pos |
| Acetylursolic acid | 499.3774287 | 14.48026667 | pos |
| Benzylideneacetone | 147.080345 | 2.986366667 | pos |
| 1-O-Acetylbritannilactone | 309.1666177 | 5.824933333 | pos |
| 2-Methylbenzoic acid | 137.059521 | 5.028516667 | pos |
| Methyl lucidenate Q | 533.3115121 | 8.257316667 | neg |
| Bisphenol A | 229.1219147 | 7.712466667 | pos |
| Vanillyl alcohol | 135.0437595 | 3.961066667 | neg |
| Ethylmethylmaleimide | 140.0705124 | 3.023466667 | pos |
| Ceplignan | 327.0869491 | 7.183433333 | neg |
| L-Asparagine | 131.0447545 | 0.591516667 | neg |
| (-)-Caryophyllene oxide | 221.1895522 | 6.864583333 | pos |
| Capillanol | 175.1115323 | 6.499433333 | pos |
| Valerophenone | 163.1114952 | 6.39335 | pos |
| Trans,trans-2,4-Heptadienal | 111.0806307 | 3.17455 | pos |
| Indene | 117.0699522 | 3.909233333 | pos |
| Swertiamarin | 339.1069083 | 5.949766667 | pos |
| Nootkatone | 219.1740177 | 5.970433333 | pos |
| Cathinone | 132.0807902 | 3.3281 | pos |
| Ajmalicine | 373.1500753 | 4.57805 | neg |
| Secoxyloganic acid | 389.1086546 | 2.641216667 | neg |
| 3,4-Dimethylstyrene | 133.1010798 | 3.6465 | pos |
| Laminine | 189.159498 | 0.545566667 | pos |
| Officinalic acid | 499.3061228 | 11.62155 | neg |
| 6-methyluracil | 127.0500842 | 2.066983333 | pos |
| Tremetone | 203.1063949 | 4.186333333 | pos |
| 3,4-Bis(methoxycarbonyl)benzoic acid | 237.039792 | 4.57805 | neg |
| Gamma-Linolenic acid | 279.2315555 | 12.22443333 | pos |
| Allyl hexanoate | 121.1011163 | 3.628583333 | pos |
| 9-oxononanoic acid | 171.1013124 | 6.985933333 | neg |
| Pantothenic acid | 218.1025145 | 2.365433333 | neg |
| Aromatic aldehyde | 107.0494119 | 2.0875 | pos |
| Curcumenolactone C | 265.1436433 | 3.005066667 | pos |
| Beta-Elemonic acid | 437.3408563 | 13.52405 | pos |
| 3-Epicorosolic acid | 514.3887894 | 13.5896 | pos |
| 5,7,3'-Trihydroxy-6,4',5'-trimethoxyflavone | 361.0911573 | 7.973516667 | pos |
| Alisol C 23-acetate | 527.3373861 | 12.38266667 | neg |
| Cucurbitacin B | 559.3258181 | 11.5513 | pos |
| Guanosine | 282.0839888 | 1.140666667 | neg |
| Alpha-cyperone | 219.1740266 | 6.758283333 | pos |
| Jioglutin E | 231.1229802 | 4.7499 | neg |
| Indole-3-carboxaldehyde | 146.0598934 | 4.683566667 | pos |
| (-)-Senkyunolide J | 244.1540832 | 3.387666667 | pos |
| Chavicol | 135.0802516 | 6.9881 | pos |
| Toddalolactone | 291.1220667 | 10.0164 | pos |
| 2,6-Dimethoxy-benzoic acid | 181.0494722 | 6.339966667 | neg |
| Meso-dihydroguaiaretic acid | 331.1873217 | 8.262166667 | pos |
| Myrianthic acid | 549.3426953 | 10.54255 | neg |
| Pseudoionone | 175.1478782 | 6.864583333 | pos |
| Benzoic acid | 123.0439925 | 6.9881 | pos |
| Lathyrol | 335.2188758 | 11.97788333 | pos |
| Isovanillic acid | 169.0494303 | 3.367483333 | pos |
| Alpha-Cyclogeraniol acetate | 228.1954068 | 9.39725 | pos |
| Beta-Calacorene | 201.1634609 | 8.306783333 | pos |
| Piperonylic acid | 165.0180899 | 3.353116667 | neg |
| Sorbic acid | 95.04951898 | 3.136516667 | pos |
| Jasmone | 147.1165908 | 6.864583333 | pos |
| Gamma-Calacorene | 201.1636887 | 12.09075 | pos |
| 3-n-Butylphathlide | 191.1064521 | 4.2427 | pos |
| Vitetrifolin F | 365.2681027 | 13.98458333 | pos |
| Isovanillin | 151.0387421 | 3.332266667 | neg |
| Auraptene | 297.1523811 | 13.86733333 | neg |
| Ketoleucine | 189.0757682 | 4.039716667 | neg |
| Qing Hau Sau | 283.1534301 | 5.7407 | pos |
| Alpha-Calacorene | 201.1635057 | 9.528733333 | pos |
| Glyceryl palmitate | 313.2731643 | 14.2534 | pos |
| Anwulignan | 329.1743466 | 12.02313333 | pos |
| Teucrol | 317.1013151 | 5.36895 | pos |
| (+/-)-Camphor | 135.1168016 | 3.2509 | pos |
| 2-Adamantanone | 151.1115085 | 4.969933333 | pos |
| 2,3,4-trihydroxybenzoic acid | 153.0180878 | 6.073533333 | pos |
| Stearidonic Acid | 277.2158794 | 11.39503333 | pos |
| Phthalic anhydride | 149.0232949 | 13.45566667 | pos |
| 1,5-dimethyl-1-vinyl-4-hexenyl acetate | 161.132389 | 12.09075 | pos |
| 4-Hydroxycinnamamide | 146.0599143 | 3.9272 | pos |
| L-Aspartic acid | 132.0288128 | 3.698783333 | neg |
| Methyl gallate | 183.0287517 | 3.135183333 | neg |
| Hexadecanedioic acid | 291.1950313 | 13.05241667 | pos |
| [8]-Paradol | 307.226168 | 10.5165 | pos |
| Epomediol | 185.1171531 | 5.280683333 | neg |
| Galbacin | 341.1377026 | 6.499433333 | pos |
| 5-hydroxy-7-(4-hydroxy-3-methoxyphenyl)-1-phenylheptan-3-one | 329.1742541 | 11.75391667 | pos |
| Darutigenol | 345.2417521 | 8.6328 | pos |
| Chiisanogenin | 543.3320446 | 13.33416667 | neg |
| Moracin M | 241.0499114 | 6.183966667 | neg |
| Sphinganine | 302.3048914 | 12.44926667 | pos |
| 4-Methoxyphenylacetic acid | 165.0544568 | 4.57805 | neg |
| Plastoquinone-1 | 205.1220944 | 5.8037 | pos |
| 3',4',5'-O-Trimethyltricetin | 345.0961656 | 7.426566667 | pos |
| Eudesmic acid | 211.060203 | 5.2374 | neg |
| Senkyunolide G | 209.1169457 | 5.409116667 | pos |
| Machilin D | 367.1511665 | 11.46188333 | pos |
| Shikimate | 173.0443663 | 3.173783333 | neg |
| 3-Methoxybenzoic Acid | 135.0439516 | 5.8463 | pos |
| Ilexgenin A | 561.3427514 | 12.22636667 | neg |
| Maslinic acid | 471.3473344 | 13.71053333 | neg |
| (+)-Ganodermanondiol | 439.3563237 | 15.0692 | pos |
| 5,7-Dihydroxychromone | 177.0181777 | 4.557716667 | neg |
| Butyl p-hydroxybenzoate | 258.1119553 | 7.626183333 | pos |
| Dihydroartemisinic acid | 237.1846047 | 11.5513 | pos |
| Sesamol | 121.0285225 | 2.9487 | pos |
| 2-Methoxycinnamaldehyde | 163.0752355 | 12.09075 | pos |
| Neocnidilide | 195.1377412 | 9.528733333 | pos |
| 4'-Demethylepipodophyllotoxin | 399.1078017 | 9.575 | neg |
| 4-Methylcatechol | 125.0597357 | 4.30095 | pos |
| Lunularic acid | 257.0813578 | 8.21275 | neg |
| Menthofuran | 133.1010718 | 5.638066667 | pos |
| 2-phenylethylamine | 122.0964798 | 2.794183333 | pos |
| 7-hydroxycoumarine | 161.0230864 | 4.834416667 | neg |
| Crocetin | 327.1596543 | 12.0029 | neg |
| Glutamic Acid | 112.0394977 | 3.194233333 | pos |
| Cleroindicin B | 203.0914935 | 3.698783333 | neg |
| Quillaic acid | 485.326733 | 13.33416667 | neg |
| M-Xylene | 107.0857006 | 5.99135 | pos |
| Ganoderic acid C2 | 499.3062536 | 12.2935 | neg |
| Eucommiol | 153.0907059 | 13.85673333 | pos |
| Axillarin | 345.0610477 | 6.030016667 | neg |
| Rishitin | 223.1688752 | 6.415266667 | pos |
| Acetovanillone | 165.0544198 | 5.612266667 | neg |
| Salicylic acid | 139.0388134 | 5.327133333 | pos |
| 4,7-Dimethyl-1-tetralone | 175.1114821 | 9.007733333 | pos |
| Roughanic acid | 251.2000202 | 9.35305 | pos |
| Guaiazulene | 199.147895 | 9.462366667 | pos |
| Sedanonic acid lactone | 193.1221227 | 9.419216667 | pos |
| 2-Hydroxyquinoline | 144.044133 | 4.686 | neg |
| 4-isopropylbenzoic acid | 147.0802106 | 13.85673333 | pos |
| Pimelic acid | 159.0649652 | 3.980566667 | neg |
| Dihydrocarvone | 153.1271985 | 3.445966667 | pos |
| Carnosic acid | 331.1910657 | 8.0574 | neg |
| Licoleafol | 353.1024998 | 5.678866667 | neg |
| Mevalonic acid | 113.0598164 | 3.835166667 | pos |
| Aurantiamide | 401.1863376 | 10.00243333 | neg |
| Eriodictyol | 287.0556674 | 5.7011 | neg |
| Ricinoleic acid | 297.2428396 | 13.31221667 | neg |
| Medicagenic acid | 561.3426395 | 12.71768333 | neg |
| 2-oxindole | 134.0599649 | 5.409116667 | pos |
| O-Xylene | 107.0856192 | 6.9881 | pos |
| Hispidone | 471.3472386 | 13.09181667 | neg |
| Cuminyl acetate | 193.1222362 | 12.9849 | pos |
| Nortricycloekasantalic acid | 181.1220603 | 6.39335 | pos |
| Aromadendrane-4,10-diol | 221.1895646 | 9.246016667 | pos |
| Myristic acid | 246.2422728 | 8.1959 | pos |
| Jasmonic acid | 211.1325609 | 9.617716667 | pos |
| Grandifloric acid | 363.214749 | 13.13606667 | neg |
| Maltose | 323.0979458 | 4.7285 | neg |
| Cycloastragenol | 532.3986759 | 14.36663333 | pos |
| Guaiacol | 125.0596535 | 7.648283333 | pos |
| Blumenol B | 209.1533076 | 14.32168333 | pos |
| Ketoisophorone | 151.0750512 | 5.790133333 | neg |
| Osthole | 245.1169559 | 12.42675 | pos |
| N-benzoyl-l-phenylalaninol | 256.1327591 | 6.80105 | pos |
| 2-buten-1-one, 1-(2,6,6-trimethyl-1,3-cyclohexadien-1-yl)- | 191.1427113 | 9.007733333 | pos |
| Piperonyl alcohol | 135.0439327 | 6.521016667 | pos |
| 5,7-dihydroxyflavanone | 255.0656707 | 10.25008333 | neg |
| 9-Hydroxynonanoic acid | 173.1170116 | 8.725516667 | neg |
| Longicamphenylone | 207.1741777 | 11.35035 | pos |
| 4-n-Hexylphenol | 179.1428817 | 13.25543333 | pos |
| 11-Hydroxy-sugiol | 361.1992284 | 12.82915 | neg |
| Mannosamine | 162.0758715 | 0.526883333 | pos |
| Caprylic acid | 189.1121202 | 5.590183333 | neg |
| 6-Methyl-7-(3-oxobutyl)-bicyclo[4.1.0]heptan-3-one | 195.1378788 | 12.38146667 | pos |
| 10-hydroxy-2e-decenoic acid | 231.1230431 | 5.172433333 | neg |
| 3,7-dimethyloct-6-enal | 199.1328985 | 6.228216667 | neg |
| Stigmasterol | 413.3770617 | 15.41041667 | pos |
| 5-O-Methylembelin | 307.1910589 | 8.747783333 | neg |
| 4-n-Pentylphenol | 165.1271941 | 10.86673333 | pos |
| P-Anisic Acid | 153.0543419 | 7.20975 | pos |
| Fa(9:0) | 203.1277982 | 7.183433333 | neg |
| (-)-gamma-ionone | 193.1585026 | 14.32168333 | pos |
| Bis(2-ethylhexyl) phthalate | 391.2836252 | 15.16026667 | pos |
| Geraniol | 199.1328764 | 6.720466667 | neg |
| Mayurone | 205.1585251 | 12.92023333 | pos |
| Beta-Hydroxypropiovanillone | 197.0805336 | 9.0508 | pos |
| 2-hydroxyhexadecanoic acid | 271.2273763 | 14.0918 | neg |
| Isorosmanol | 327.1596288 | 12.69511667 | neg |
| Dictamnol | 179.1428799 | 11.2406 | pos |
| 9,10-Dihydroxystearate | 315.2533171 | 12.87386667 | neg |
| Icosatrienoic acid | 324.2892448 | 13.72518333 | pos |
| Monobutyl phthalate | 221.0808707 | 7.339516667 | neg |
| L-histidine | 154.0608379 | 0.591516667 | neg |
| Geranyl acetate | 195.1378848 | 9.530216667 | neg |
| Sedanonic acid | 209.1172074 | 9.418166667 | neg |
| Anisole | 91.05453725 | 13.85673333 | pos |
| Fa(10:0) | 217.143556 | 9.039616667 | neg |
| Esculetin | 177.0180519 | 6.787066667 | neg |
| 6-methylhept-5-en-2-ol | 111.1169324 | 13.77088333 | pos |
| Parthenicin | 261.1126733 | 10.67723333 | neg |
| Phthalic acid | 167.0336398 | 15.16026667 | pos |
